# Analyzing the dominant SARS-CoV-2 transmission routes toward an *ab initio* disease spread model

**DOI:** 10.1063/5.0034032

**Published:** 2020-12-01

**Authors:** Swetaprovo Chaudhuri, Saptarshi Basu, Abhishek Saha

**Affiliations:** 1 Institute for Aerospace Studies, University of Toronto, Toronto, Ontario M3H 5T6, Canada; 2 Department of Mechanical Engineering, Indian Institute of Science, Bengaluru, KA 560012, India; 3 Department of Mechanical and Aerospace Engineering, University of California San Diego, La Jolla, California 92093, USA

## Abstract

Identifying the relative importance of the different transmission routes of the SARS-CoV-2 virus is an urgent research priority. To that end, the different transmission routes and their role in determining the evolution of the Covid-19 pandemic are analyzed in this work. The probability of infection caused by inhaling virus-laden droplets (initial ejection diameters between 0.5 *µ*m and 750 *µ*m, therefore including both airborne and ballistic droplets) and the corresponding desiccated nuclei that mostly encapsulate the virions post droplet evaporation are individually calculated. At typical, air-conditioned yet quiescent indoor space, for average viral loading, cough droplets of initial diameter between 10 *µ*m and 50 *µ*m are found to have the highest infection probability. However, by the time they are inhaled, the diameters reduce to about 1/6th of their initial diameters. While the initially near unity infection probability due to droplets rapidly decays within the first 25 s, the small yet persistent infection probability of desiccated nuclei decays appreciably only by O(1000s), assuming that the virus sustains equally well within the dried droplet nuclei as in the droplets. Combined with molecular collision theory adapted to calculate the frequency of contact between the susceptible population and the droplet/nuclei cloud, infection rate constants are derived *ab initio*, leading to a susceptible-exposed-infectious-recovered-deceased model applicable for any respiratory event–vector combination. The viral load, minimum infectious dose, sensitivity of the virus half-life to the phase of its vector, and dilution of the respiratory jet/puff by the entraining air are shown to mechanistically determine specific physical modes of transmission and variation in the basic reproduction number $R_{0}$ from first-principles calculations.

## I. INTRODUCTION

One of the longstanding questions of pandemics involving respiratory droplets is identifying their dominant mode of transmission. The most well recognized pathways for infectious respiratory diseases are (i) the direct contact/inhalation of the relatively larger infectious droplets (>5 *µ*m) commonly known as the droplet mode of transmission, (ii) airborne or aerosol transmission, which is presumed to be caused by inhalation of very small infectious droplets (<5 *µ*m) floating in air, and (iii) contact with infectious surfaces–fomites. For the present Covid-19 pandemic, while the droplet mode of transmission is well established, evidence for aerosol transmission^1,2^ renders identifying the dominant transmission route an intriguing scientific problem with extremely high implications for human health and public policy. On July 9, 2020, the World Health Organization issued a scientific brief^3^ stating “Urgent high-quality research is needed to elucidate the relative importance of different transmission routes; the role of airborne transmission in the absence of aerosol generating procedures.” In this paper, we establish a fundamental theoretical framework where the relative strength of the individual transmission routes is analyzed from first-principles with idealizing assumptions. Many biological aspects of the disease transmission including but not limited to effects of immune response are beyond the scope of this paper and will not be addressed here with an exclusive focus on the physical aspects^4–10^ of the disease transmission. Physics is involved in at least four levels in a Covid-19 type pandemic evolution: micro-scale droplet physics, spray/droplet-cloud physics, collision/interaction between the spray/cloud and the susceptible individuals, and deposition and absorption of the inhaled droplets/droplet nuclei. The first three are addressed in this paper at different levels of complexity. We adopt the convention that respiratory droplets (all liquid phase droplets of all sizes, typically 0.5 *µ*m–750 *µ*m, including both airborne and ballistic droplets) cause disease transmission by “droplet or *d*” route, whereas dried or desiccated droplet nuclei, which, in this paper, refer to the semi-solid/crystalline residue that remains after the droplet liquid evaporates, are responsible for the “dried droplet nuclei or *n*” route of transmission. Thus, the *d* route invariably includes droplets less than as well as greater than 5 *µ*m, instead of resorting to the rather arbitrary threshold to distinguish between droplets and nuclei. The reason of our choice is that the distinct thermodynamic phase of the transmission vector (liquid vs solid/semi-solid) is expected to be a much better identifier to delineate the different pathways. Furthermore, the virus survivability within the dried droplet nuclei could be well different from that of the liquid droplet. Small and medium sized droplets do remain airborne after their ejection for substantially long periods of time^11^ due to the fact that the droplet size continuously changes, except in highly humid conditions (*RH*_*∞*_ > 85%), due to evaporation. Respiratory droplets are ejected during different expiratory events: breath, cough, sing, sneeze, or talk(ing), when the droplets are ejected with different droplet size distributions.^12–14^ In violent expiratory events like coughing or sneezing, the droplets co-move with a turbulent jet of exhaled air. The trajectories of the jet and the droplets could diverge due to droplet inertia and gravity effects. Nevertheless, experiments by Bourouiba *et al.*^15,16^ have shown that these droplets can travel rather large distances initially within a turbulent jet, which later transition to a puff or a cloud due to the lack of a continuous momentum source. Depending on the ambient conditions and droplet size, these droplets evaporate at different times. However, while water, the volatile component of the mucosalivary liquid, evaporates, the non-volatile components, salt, protein, mucus, and virus particles present, separate out by crystallization processes. It is to be noted that a homogeneous solution droplet without any admixture or impurities undergoes homogeneous nucleation toward salt crystallization only below a certain threshold relative humidity called efflorescence relative humidity, which is 45% for NaCl at room temperatures.^17^ However, in the presence of different components, especially 100 nm sized virus particles, there is no dearth of nucleation sites in infectious mucosalivary liquid. Hence, in this paper, we will consider salt crystallization beyond a certain threshold supersaturation to form droplet residues at all relative humidities. These droplet residues, typically about 10%–20% of the initial droplet diameter are called dried droplet nuclei, remain floating as aerosols and are believed to be responsible for the airborne mode of droplet transmission. While only very few experiments have so far probed the structure of these dried droplet nuclei, the first of its kind work by Vejerano and Marr^18^ provided critical insights on the distribution of virus particles inside the dried droplet nuclei. Marr *et al.*^19^ offered mechanistic insights on the role of relative humidity in respiratory droplet evaporation but the question on the role of evaporation, the resulting chemistry inside the dried droplet nuclei on virus survivability persisted. This was explored by Lin and Marr.^20^ It was found that virus survivability inside sessile droplets is a non-monotonic function of ambient relative humidity and of course dependent on the specific virus type as well. Questions on the survivability of the SARS-CoV-2 virus inside the dried droplet nuclei from contact free droplets, as it would happen for respiratory sprays, are not yet settled. In this paper, first, we present a model to identify the probability of infection transmission for two different routes *d* and *n* by accounting for the corresponding droplet size distribution, viral load, and virus half-life. Next, we briefly present the droplet/nuclei cloud aerodynamics and respiratory droplet evaporation physics. The 1% (w/w) NaCl–water solution is used as a surrogate for the mucosalivary fluid. This is followed by modeling the generalized infection rate constant, which can be used in theory for any kind of expiratory event or for any mode of transmission. The rate constant is then incorporated into a Susceptible-Exposed-Infectious-Recovered-Deceased (SEIRD) model^21^ using the formalism of a chemical reaction mechanism. Finally, the Results and Discussion are presented followed by the Conclusions.

At present, widely used epidemiological models for infectious respiratory diseases do not account for the underlying flow physics of disease transmission. In most epidemiological models, the rate constants (parameters of the SEIRD differential equations) that lead to $R_{0}$ are obtained by fitting available data on the number of new infections.^21,22^ Indeed, these types of epidemiological models have provided immense insights on Covid-19 and the necessity of non-pharmaceutical public health interventions.^22–24^ However, it is to be recognized that the data for a Covid-19 type pandemic are almost always under-reported due to a large number of asymptomatic cases. Furthermore, the actual rate constants and $R_{0}$ could depend on several physical factors such as temperature, relative humidity, UV-index, and so on. Thus, with changing conditions, parameters obtained from fitting recent past data may not be adequate, standalone, to predict the nature of future outbreaks. Therefore, there is a pressing need to develop a framework to understand and calculate $R_{0}$ from *ab initio* calculations while being cognizant of the idealizing assumptions and limitations involved in the process. To the best of the authors’ knowledge, this is the first time that the aerodynamics and thermodynamics of the droplets/nuclei, viral load, half-life, and minimum required viral dose for infection have been systematically accounted for to obtain the rate constants and $R_{0}$ of a SEIRD model, *ab initio*.

## II. THE MODEL

### A. Probability of infection by different transmission routes

**FIG. 1.**
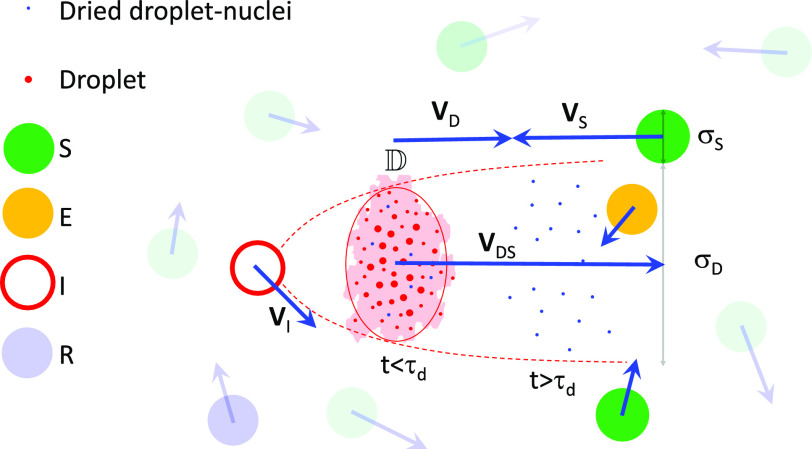
Schematic of the interaction between *S* and the droplet/dried droplet nuclei cloud D ejected by *I*, resulting in *E*. The red ellipse marks the control volume containing the turbulent cloud (colored by red) on average, analyzed for computing the probability of infection $P_{\alpha \beta }$.

In this subsection, we estimate the probability of infection by different transmission modes for any expiratory event. Consider an infected person *I* exhaling a droplet laden jet that quickly transforms into a droplet cloud D in the vicinity of susceptible individuals *S*, as shown in Fig. 1.

The instantaneous diameter of the respiratory jet/puff/cloud is given by *σ*_*D*_, its velocity with respect to *S* is given by ${\vec{V}}_{DS}$, while the effective diameter of the hemispherical volume of air inhaled by *S*, for every breath, is given by *σ*_*S*_. *σ*_*DS*_ = (*σ*_*D*_ + *σ*_*S*_)/2. The primary objective of this subsection is to estimate $P_{\alpha \beta }\left(t\right)$, which denotes the time dependent probability of infection of *S* for the given expiratory event *α* and the type of transmission vector *β*. Thus, *α* denotes one among breathing, coughing, singing, sneezing, or talking, while *β* denotes one among droplets, dried droplet nuclei, or fomites. It is to be recognized that $\sigma_{DS},{\vec{V}}_{DS}$ are not only functions of time but also dependent on *α* and *β*, though their subscripts have been dropped for brevity. As the droplet/nuclei cloud entrains surrounding air, it grows in size with concomitant dilution of the particles inside, thereby reducing $P_{\alpha \beta }$. Of course, $P_{\alpha \beta }$ must also be determined by the viral load and droplet size distribution. $P_{\alpha \beta }$ could be obtained by solving the transport equations. Instead, here we take a Lagrangian approach of tracking and analyzing the control volume of the air-droplet cloud D ejected by *I* and droplets/droplet nuclei within. At the moment of the onset of the expiratory event denoted by *t* = 0, a log-normal distribution could be used to describe the probability density function (pdf) of the initial droplet size distribution *f*_*α*_ of the ejected respiratory spray,

$$f_{\alpha }\left(D\right)=\frac{1}{\sqrt{2\pi }\sigma D}e^{-{\left(\ln\left(D\right)-\mu \right)}^{2}/2\sigma^{2}},$$ (1)

where *D* is the sample space variable of the initial droplet diameter *D*_*s*,0_, and *μ* and *σ* are the mean and standard deviation of ln(*D*), respectively. If *N*_*tα*_ is the total number of droplets ejected for the expiratory event *α*, the number of droplets within the interval *dD* is given by *N*_*tα*_*f*_*α*_(*D*)*dD*. Therefore, for a given *f*_*α*_ and *ρ*_*v*_, the viral load in the number of copies per unit volume of the ejected liquid, the cumulative number of virions in droplets between sizes *D*_1_ and *D*_2_ is given by

$$N_{v\alpha }=\frac{\pi \rho_{v}N_{t\alpha }}{6}\int_{D_{1}}^{D_{2}}D^{3}f_{\alpha }\left(D\right)dD.$$ (2)

For the SARS-CoV-2 virus, Wölfel reported^25^ the average viral load in sputum to be *ρ*_*v*_ = 7 × 10^6^ copies/ml, while the maximum is given by *ρ*_*v*,*max*_ = 2.35 × 10^9^ copies/ml. Utilizing Eq. (2), we can define $N_{\alpha d}\left(t\right)$ (time dependent number of virions inhaled from droplets). For an infection to occur, some non-zero number of active virions must be found in the droplets present in the total volume of air inhaled. The maximum time for *S* to cross the volume with diameter *σ*_*DS*_ is given by *t*_*cross*_ = (*σ*_*S*_ + *σ*_*D*_)/*V*_*DS*_, while the number of breaths per unit time is *N*_*b*_ ≈ 16/60 s^−1^^26^ and the volume inhaled per breath is $V_{b}=\left(4/6\right)\pi \sigma_{S}^{3}$. Therefore, the total volume of air inhaled while crossing the respiratory cloud is $V_{a}=\left(4/6\right)\pi N_{b}\sigma_{S}^{3}\left(\sigma_{S}+\sigma_{D}\right)/V_{DS}$. The fraction of virion population surviving within the droplets or dried-droplet nuclei at time *t* is given by *ψ*_*β*_(*t*) and can be assumed to decay as $\psi_{\beta }\left(t\right)={\left(1/2\right)}^{t/t_{\beta \frac{1}{2}}}$. $t_{\beta \frac{1}{2}}$ is the half-life of the SARS-CoV-2 virus in *d* or *n*. While the half-life of the SARS-Cov-2 within aerosols, in general, could be estimated from Refs. 27 and 28, the distinction of the half-life of the virus within airborne droplets or dried droplet nuclei is not yet available to our knowledge. While this information is indeed most critical, in view of its absence, for the present work, we will mostly assume *ψ*_*d*_(*t*) = *ψ*_*n*_(*t*) = *ψ*(*t*) and $t_{\beta \frac{1}{2}}=t_{\frac{1}{2}}$, unless specifically mentioned. At typical indoor conditions, *T*_*∞*_ = 21.1 °C and *RH*_*∞*_ = 50% and with UV index =1 on a scale of 10, $t_{\frac{1}{2}}=15.25$ min.^27,28^ Accounting for these, $N_{\alpha d}\left(t\right)$ is given by the following equation:

$$\begin{array}{ccc} N_{\alpha d}\left(t\right) & = & \frac{\pi \rho_{v}N_{t\alpha }N_{b}\sigma_{S}^{3}\left(\sigma_{S}+\sigma_{D}\left(t\right)\right)\psi_{d}\left(t\right)}{12V_{DS}\left(t\right)\sigma_{D}^{3}\left(t\right)}\\ & & \times \;\int_{D_{1}\left(t\right)}^{D_{2}\left(t\right)}D^{3}f_{\alpha }\left(D\right)dD. \end{array}$$ (3)

**FIG. 2.**
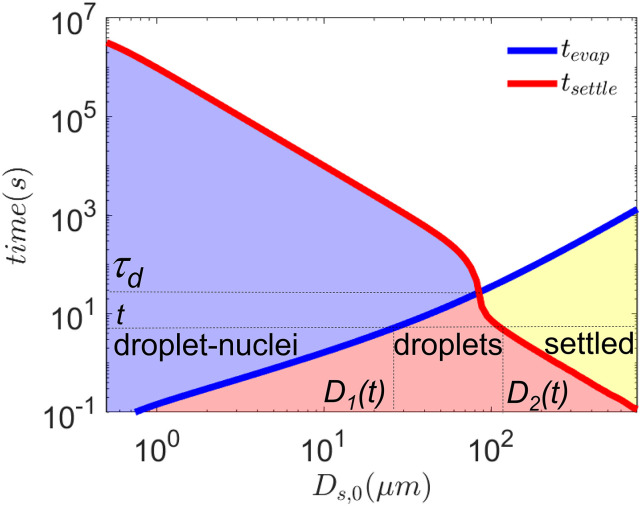
Wells curves modified by accounting for droplet cloud aerodynamics, thermodynamics, and droplet desiccation for 1% NaCl–water droplets at *T*_*∞*_ = 21.44 °C and *RH*_*∞*_ = 50%. After time *t*, the droplets with initial size *D*_*s*,0_ < *D*_1_(*t*) have been converted to dried droplet nuclei; those within *D*_1_(*t*) ≤ *D*_*s*,0_ ≤ *D*_2_(*t*) are in the liquid droplet state; *D*_*s*,0_ > *D*_2_(*t*) have settled and could be potential fomites. *τ*_*d*_ = 22.87 s. The red, blue, and yellow shaded regions denote the regimes of droplets, dried droplet nuclei, and fomites, respectively.

Here, *D*_1_(*t*) and *D*_2_(*t*) are the minimum and maximum of initial droplet diameters, respectively, available in the droplet cloud after time *t*, as shown in Fig. 2. The non-linearity of *t*_*settle*_ vs *D*_*s*,0_ (in the log–log plot of Fig. 2) occurs due to the phase transition of the droplet population. Beyond *τ*_*d*_, all droplets have been converted into dried droplet nuclei. At time *t*, droplets with *D*_*s*,0_ < *D*_1_(*t*) have evaporated and have been converted to dried droplet nuclei, while *D*_*s*,0_ > *D*_2_(*t*) have escaped by gravitational settling and have been converted to potential fomites. Clearly, as *σ*_*D*_ increases with time, $N_{\alpha d}$ decreases due to dilution effects and also because droplet numbers are depleted by evaporation and settling. Since the maximum evaporation time of the airborne droplets $\tau_{d}\ll t_{\beta \frac{1}{2}}$, the effect of virus half-life on $N_{\alpha d}$ is negligible. The details of the methodology to derive the parameters concerning the respiratory jet and droplet dynamics from the conservation principles of mass, momentum, energy, and species could be found briefly in Subsections II B and II C, respectively. Further details could be found in the work of Chaudhuri *et al.*^29^

It is essential to note that in many diseases, uncertainty exists over transmission routes. For example, in the case of Covid-19, the transmission by dried droplet nuclei is not certain. As such, we do not know for sure if the SARS-CoV-2 virus survives within dried droplet nuclei and remain infectious, though there is evidence that SARS-CoV-2^30^ and some other virus do survive quite well inside dried droplet nuclei.^19^ Even if they do, their half-life and infection potential could be different with respect to those inside droplets. Thus, as would be shown later, it is essential to define different rate constants for different transmission modes. Since the dried droplet nuclei are a product of the droplets, itself, the two routes are highly coupled and are not independent.

For the droplet nuclei, $N_{\alpha n}$is given by

$$\begin{array}{ccc} N_{\alpha n}\left(t\right) & = & \frac{\pi \rho_{v}N_{t\alpha }N_{b}\sigma_{S}^{3}\left(\sigma_{S}+\sigma_{D}\left(t\right)\right)\psi_{n}\left(t\right)}{12V_{DS}\left(t\right)\sigma_{D}^{3}\left(t\right)}\\ & & \times \;\int_{0}^{D_{n}\left(t\right)}D^{3}f_{\alpha }\left(D\right)dD, \end{array}$$ (4)

*D*_*n*_(*t*) = *D*_1_(*t*) if *t* < = *τ*_*d*_, and *D*_*n*_(*t*) = *D*_2_(*t*) if *t* > *τ*_*d*_. $N_{\alpha n}\left(t\right)$ decreases with time due to the increase in *σ*_*D*_ with time, i.e., the dilution effect as well due to virus half-life. $N_{\alpha n}\left(t\right)$ decreases with time due to the increase in *σ*_*D*_ with time, i.e., the dilution effect as well due to virus half-life.

The generalized probability of infection $P_{\alpha \beta }$ as a function of infectious dose $N_{\alpha \beta }$ inhaled while crossing the droplet/nuclei cloud D, can now be expressed as

$$P_{\alpha \beta }\left(t\right)=1-e^{-r_{v}N_{\alpha \beta }\left(t\right)}.$$ (5)

The total probability of infection for the expiratory event *α* could be defined as

$$P_{\alpha }\left(t\right)=1-e^{-r_{v}\sum_{\beta }N_{\alpha \beta }\left(t\right)}.$$ (6)

The form of Eq. (6) is based on the dose response model by Haas,^31^ which has been used by Nicas,^32^ Sze To *et al.*,^33^ and many other authors to calculate the infection probability. Mathematically, it is also similar to the Wells–Riley equation^34^ used by Buonanno *et al.*^35^ to assess the aerosol risk of SARS-CoV-2 during talking and breathing. However, in contrast to these works, here, droplet cloud aerodynamics (Subsection II B) coupled with the detailed droplet evaporation-nuclei production mechanism (Subsection II C) and droplet settling dynamics are utilized in a semi-analytical framework to calculate the time varying inhaled virion number and corresponding probability of infection, probably for the first time. Eventually, as shown later, this framework will be used to calculate the basic reproduction number $R_{0}$. As such, the form of this equation is also validated by the results from the work of Zwart *et al.*,^36^ where *r*_*v*_ is a constant for a particular virus. For this paper, we will use *r*_*v*_ = 0.5 such that inhaling at least ten virions by *d* and/or *n* route would result in an infection probability $P_{\alpha \beta }\approx 1$, unless specifically mentioned. In the absence of this exact *r*_*v*_ for the SARS-CoV-2 virus at the time of writing this paper, this is an educated guess. Hence, *r*_*v*_ = 0.05 and *r*_*v*_ = 0.005 corresponding to minimum infectious doses of 100 and 1000 virions, respectively, will also be eventually explored near the end of the paper.

### B. Aerodynamics of droplets and nuclei

The droplets when ejected during respiratory events mostly follow the volume of exhaled air. Due to continuous entrainment, the exhaled air volume grows in diameter, and as a result, its kinetic energy decays with time. Bourouiba *et al.*^15^ identified that for a short duration, the exhaled droplets evolve inside a turbulent jet, which transitions to a puff at later stage. Since the respiratory droplets or the dried nuclei experience aerodynamic drag, it is essential to identify the evolution of the surrounding jet or puff. Based on the literature^37–39^ of transient turbulent jets and puffs, the following evolution equations for the axial location, velocity, and radial spread could be used:

$$\begin{array}{c} x_{j}\left(t\right)={\left(\frac{12}{K}\right)}^{1/2}{\left(U_{j,0}R_{j,0}\right)}^{1/2}t^{1/2},\\ U_{j}\left(t\right)=\frac{6U_{j,0}R_{j,0}}{Kx_{j}\left(t\right)},\\ R_{j}\left(t\right)=R_{j,0}+\left(x_{j}\left(t\right)-x_{j,0}\right)/5 \end{array}$$ (7)

and

$$\begin{array}{c} x_{pf}\left(t\right)=\left(\frac{3m}{a}\right)R_{pf}\left(t\right),\\ U_{pf}\left(t\right)=U_{pf,0}{\left(\frac{3mR_{pf,0}}{4aU_{pf,0}t}\right)}^{3/4},\\ R_{pf}\left(t\right)=R_{pf,0}{\left(\frac{4aU_{pf,0}t}{3mR_{pf,0}}\right)}^{1/4}, \end{array}$$ (8)

where the subscripts *j* and *pf* denote the jet and puff, respectively, *R*_0_ and *U*_0_ are the average radius and axial velocity at distance *x*_0_, and *K* is a characteristic constant for turbulent jet and is reported to be 0.457.^37^ At the inception of the respiratory event (*t* = 0), the jet is assumed to have a velocity of *U*_*j*,0_ = 10 m/s and a radius of *R*_*j*,0_ = 14 mm—the average radius of human mouth. For analytical tractability, we assume that all droplets of all sizes are ejected at time *t* = 0 and would not consider time variation in the ejection of the droplets. This is a safe assumption since the expiratory event like cough lasts less than a second and the turbulence of the jet and the air entrained will rapidly disperse the ejected droplets into the jet/puff in any case. The characteristic constants for puff are *a* ≈ 2.25 and *m* = (*x*_*p*,0_*a*)/(3*R*_*p*,0_).^38^ Since the continuous ejection of air from mouth lasts only for the duration of the corresponding respiratory event, the jet behavior persists only for this period. Beyond this time (≈1 s^40^), the puff behavior is observed. Hence, the velocity and the radial spread of the air surrounding the exhaled droplets will be

$$\begin{array}{c} U_{g}=\left\{\begin{array}{cc} U_{j}\left(t\right),\; & t\leq 1\text{s}\\ U_{pf}\left(t\right),\; & t>1\text{s}, \end{array}\right.\\ R_{g}=\left\{\begin{array}{cc} R_{j}\left(t\right),\; & t\leq 1\text{s}\\ R_{pf}\left(t\right)\; & t,>1\text{s}. \end{array}\right. \end{array}$$ (9)

The horizontal displacement (*X*_*p*_) of the exhaled droplet and its instantaneous velocity (*U*_*p*_) due to the drag can be solved with^41^

$$\begin{array}{c} dX_{p}/dt=U_{p},\\ dU_{p}/dt=\left(\frac{3C_{D}\rho_{v}}{8R_{s}\rho_{l}}\right)|U_{g}-U_{p}|\left(U_{g}-U_{p}\right). \end{array}$$ (10)

Here, *ρ*_*v*_ and *ρ*_*l*_ are the vapor and liquid phase densities, respectively, *R*_*s*_ is the instantaneous radius of the droplet, and *C*_*D*_ is the drag coefficient. We can assume *C*_*D*_ = 24/*Re*_*p*_ for the gas phase Reynolds number, *Re*_*p*_ = (2*ρ*_*v*_|*U*_*g*_ − *U*_*p*_|*R*_*s*_)/*μ*_*g*_ < 30.^41^ *Re*_*p*_ for the respiratory droplets are typically less than 0.1. At the time of ejection (*t* = 0) from respiratory cavities, the droplets are assumed to have a velocity (*U*_*p*,0_) close to that of the surrounding air (*U*_*j*,0_), and hence, *Re*_*p*_ ≈ 0. Thus, for *t* = 0, we use *U*_*j*,0_ − *U*_*p*,0_ = 0.01*U*_*j*,0_. The time for a droplet with initial diameter *D*_*s*,0_ [given by *t*_*settle*_(*D*_*s*,0_)] to fall a height of 1.8 m is calculated using Stokes’s settling velocity. Mathematically, *t*_*settle*_ is obtained by the following equation:

$${\left(18\;\mu \right)}^{-1}\int_{0}^{t_{settle}}\left(\rho_{l}\left(t\right)-\rho_{v}\right)gD_{s}^{2}\left(t\right)dt=h_{0}.$$ (11)

By solving Eqs. (7)–(10) over the droplet and nuclei lifetime, the axial distance traveled by them, *X*_*D*_, which is the distance of the center of the cloud, can be evaluated. As the velocity of the individual droplets approaches the surrounding gas velocity within a very short time, we assume the absolute instantaneous velocity of the droplet/nuclei cloud is given by *V*_*D*_ = *U*_*g*_ from Eq. (9). Since the droplets and nuclei are dispersed within the jet/puff, the diameter of the droplet cloud ejected by *I* can be approximated as twice the radial spread of the exhaled air, *σ*_*D*_(*t*) = 2*R*_*g*_(*t*). The puff will continue to grow in a large volume without obstruction. For a small, nearly cubic room with strong mixing and poor ventilation, a constant *σ*_*D*_ for *t* ≥ *t*_*r*_ could be estimated from the volume of the room *V*_*r*_ using $\left(4/3\right)\pi {\left(\sigma_{D}\left(t_{r}\right)/2\right)}^{3}=V_{r}$. This implies a volume filling state of the ejected cloud and a homogeneously mixed state of the aerosols. $t_{r}$ is the time at which $\sigma_{D}$ grows to $\sigma_{D}\left(t_{r}\right)$.

The exhaled volume of air is initially at a temperature (*T*_*g*,0_ = 33.25 °C) and vapor mass fraction (*Y*_1,*g*,0_ corresponding to *RH*_0_ = 71.6%) different from the ambient. The values mentioned are averaged quantities measured over several subjects according to Mansour *et al.*^42^ The instantaneous temperature and vapor mole fraction that the droplet would encounter as its own ambient are the temperature [*T*_*g*_(*t*)] and vapor mass fraction [*Y*_1,*g*_(*t*)] of this volume of air during its evolution. This can be expressed with the following scaling relation:^43^

$$\begin{array}{c} \frac{\Delta T_{g}}{\Delta T_{g,0}}=\frac{\Delta Y_{1,g}}{\Delta Y_{1,g,0}}=\frac{U_{g}}{U_{g,0}}, \end{array}$$ (12)

where Δ*T*_*g*_ = *T*_*g*_ − *T*_*∞*_ and Δ*Y*_1,*g*_ = *Y*_1,*g*_ − *Y*_1,*∞*_.

### C. Droplet evaporation

In this paper, we use 1% NaCl–water droplets as the model respiratory droplet and adopt a slightly revised evaporation model (with respect to that presented in Ref. 29) for predicting the droplet evaporation time *t*_*evap*_. It is to be recognized that the droplets are surrounded by exhaled air volume, as described in Subsection II B, and hence, it serves as the “ambient condition” for the droplet. The evaporation mass flux for quasi-steady state conditions is given by

$$\begin{array}{c} {\dot{m}}_{1}=-4\pi \rho_{v}D_{v}R_{s}ln\left(1+B_{M}\right),\\ {\dot{m}}_{1}=-4\pi \rho_{v}\alpha_{g}R_{s}ln\left(1+B_{T}\right). \end{array}$$ (13)

**FIG. 3.**
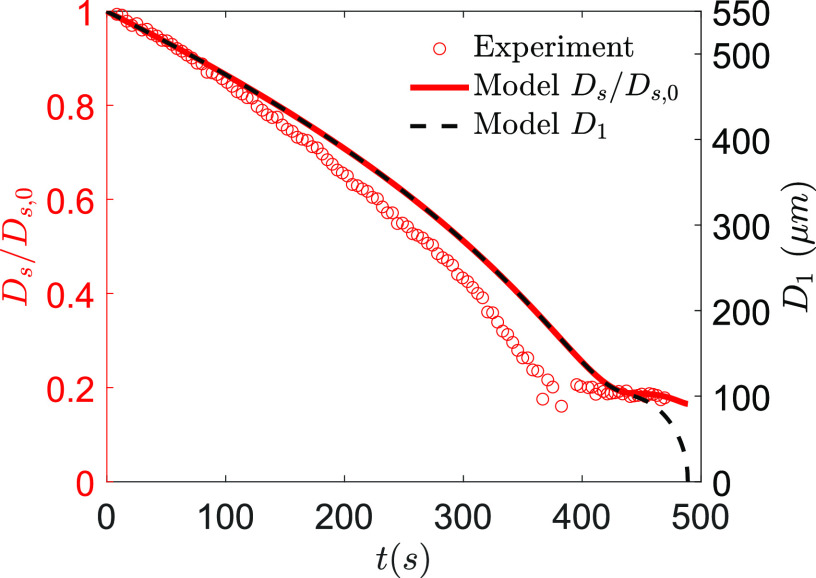
Comparison of the model output for 1% NaCl–water droplets with human saliva droplet experimental data averaged over three runs. *D*_*s*_ (normalized with *D*_*s*,0_ shown in the left ordinate) is the effective diameter of the droplet accounting for both the solute and solvent, while *D*_1_ (shown in the right ordinate) is the effective diameter only accounting for the solvent (water) mass of the droplet.

Here, ${\dot{m}}_{1}$is the droplet mass loss rate due to evaporation, *R*_*s*_ is the instantaneous droplet radius, *ρ*_*v*_ is the density of water vapor, and *D*_*v*_ is the binary diffusivity of water vapor in air, and *α*_*g*_ is the thermal diffusivity of surrounding air. *B*_*M*_ = (*Y*_1,*s*_ − *Y*_1,*g*_)/(1 − *Y*_1,*s*_) and *B*_*T*_ = *C*_*p*,*l*_(*T*_*s*_ − *T*_*g*_)/*h*_*fg*_ are the Spalding mass transfer and heat transfer numbers, respectively. *Y* is the mass fraction with the numerical subscripts 1, 2, and 3 denoting water, air, and salt, respectively. Additionally, the subscripts *s*, *g*, and *∞* denote the location at droplet surface, surrounding gas, and at very far field ambient, respectively. *h*_*fg*_ and *C*_*p*,*l*_ are the specific latent heat of vaporization and specific heat of the droplet liquid, respectively. Unlike in a pure water droplet, the vapor pressure at the surface of droplets with non-volatile dissolved substances as in respiratory droplet/salt solution droplets could be significantly suppressed. Raoult’s law provides the modified vapor pressure at the droplet surface for the binary solution, *P*_*vap*_(*T*_*s*_, *χ*_1,*s*_) = *χ*_1,*s*_*P*_*sat*_(*T*_*s*_), where *χ*_1,*s*_ is the mole fraction of evaporating solvent (here water) at the droplet surface in the liquid phase^41^ and *χ*_1,*s*_ = 1 − *χ*_3,*s*_. The far field vapor concentration, on the other hand, is related to the relative humidity of the ambient. Considering the effects of Raoult’s law and relative humidity, the vapor concentrations at the droplet surface and far field can be expressed as

$$Y_{1,s}=\frac{P_{vap}\left(T_{s},\chi_{1,s}\right)M_{1}}{P_{vap}\left(T_{s},\chi_{1,s}\right)M_{1}+\left(1-P_{vap}\left(T_{s},\chi_{1,s}\right)\right)M_{2}},$$ (14)

where *M*_1_ and *M*_2_ are the molecular weights of water and air, respectively. Instantaneous *Y*_1,*g*_ is evaluated from Eq. (12). The latent heat required for evaporation is provided by the droplet’s internal energy and/or surrounding ambient. It has been verified that the thermal gradient in the liquid phase is rather small. Therefore, neglecting the internal thermal gradients, *T*_*s*_ is obtained from the energy balance,

$$mC_{p,l}\frac{\partial T_{s}}{\partial t}=-k_{g}A_{s}\frac{\partial T_{s}}{\partial r}{|}_{s}+{\dot{m}}_{1}h_{fg},$$ (15)

where *T*_*s*_ is the instantaneous droplet temperature, $m=\left(4/3\right)\pi \rho_{l}R_{s}^{3}$ and $A_{s}=4\pi R_{s}^{2}$ are the instantaneous mass and surface area of the droplet, *ρ*_*l*_ is the density of the binary mixture of salt (if present) and water, and *k*_*g*_ is the conductivity of air surrounding the droplet. $\frac{\partial T}{\partial r}{|}_{s}$ is the thermal gradient at the droplet surface and can be approximated as (*T*_*s*_ − *T*_*g*_)/*R*_*s*_. Due to the continuous loss of water, the solution would become supersaturated in most occasions leading to the onset of crystallization. The crystallization kinetics is modeled with a one-step reaction.^44,45^ The validation of the model (1% NaCl–water solution) with saliva droplet experiments (average of three runs) from a healthy subject is shown in Fig. 3. The experiments were performed in a contact free condition in an acoustic levitator at *T*_*∞*_ = 28 °C and *RH*_*∞*_ = 41%. The reader is referred to Refs. 29 and 46 for details of the experimental configuration. Here, a difference of up to 15% could be found for the different stages of droplet drying, between the model prediction and the saliva droplet drying curve from experiments. It should be noted that human saliva contains mucus and varieties of salts and electrolytes along with compositional variations, which are difficult to model very accurately.

### D. *Ab initio* rate constants for SEIRD model

With the methodology to calculate the probability of different transmission routes identified, we proceed to evaluate the respective “rate constants.” Widely used epidemiological models do not account for the flow physics of disease transmission. Within the framework of the well known SIR-model, the model constants proposed by Stilianakis and Drossinos^47^ included overall droplet cloud features like the number of droplets per unit volume of the cloud but did not include crucial physics like cloud aerodynamics, evaporation, or crystallization dynamics that lead to droplet-nuclei formation. As such, these control the time evolution of the droplet cloud, and as shown later, the spatio-temporal evolution of the cloud and the constituent droplets plays a major role in determining the critical rate constants of the problem. A model connecting the macro-scale pandemic dynamics with the micro-scale droplet physics accounting for droplet-cloud aerodynamics, evaporation, and crystallization physics has been recently presented by Chaudhuri *et al.*^29^ Drawing inspiration from the well known molecular collision theory of reactions due to collisions, a chemical reaction mechanism was obtained where three elementary reactions described the pandemic evolution. Adopting the notations of the SEIRD model, one of the reaction rate constants that determined the conversion of a susceptible individual *S* to an exposed individual *E*, upon contact with the droplet cloud D ejected by the infectious person *I*, was denoted by *k*_1_ (or *k*_1,*o*_ as opposed to the new rate constant to be defined here) and was called infection rate constant. This *k*_1,*o*_ was modeled using the molecular collision theory.^48^ From Chaudhuri *et al.*,^29^ the expected number of collisions per unit time between *S* and D of *I* is given by $\pi \sigma_{DS}^{2}V_{DS}n_{I}n_{S}$ resulting in the infection reaction *I* + *S* → *I* + *E*. *n*_*I*_ and *n*_*S*_ are the number of infected *I* and susceptible *S* people in unit volume. The infection rate constant of this reaction is then given by

$$k_{1,o}=\pi n_{total}\sigma_{DS}^{2}V_{DS}\left(\tau_{d}/t_{c}\right).$$ (16)

*σ*_*DS*_ is the jet/puff diameter, which is also assumed to be the diameter of the droplet cloud. ${\vec{V}}_{DS}$ is the relative velocity of the droplet cloud D with respect to *S*, while *τ*_*d*_ is the droplet lifetime. *V*_*S*_ can be approximated as the preferred walking speed, which according to Refs. 49 and 50 equals to 1.3 ± 0.3 m/s, and hence, for the current study, we will assume *V*_*S*_ = 1.3 m/s. *t*_*c*_ is the average time period between two expiratory events. *t*_*c*_ is calculated as *t*_*c*_ = 3600 × 24/*N*_exp_ where *N*_exp_ is the average number of infecting expiratory events per person per day. We assumed *N*_*exp*_ = 3 from the measured coughing frequency of 0–16 in normal subjects.^51^

Equation (16) is limited by several simplifying assumptions. In this paper, we develop a comprehensive model beyond these limitations, which is also rendered capable of delineating the relative dominance of the different disease transmission pathways. In particular, in the following, we derive a new rate constant accounting for (i) the finite viral loads, finite viral lifetime, and the corresponding probability of infection, (ii) the evolution of the collision volume with time, (iii) transmission by droplets of any sizes and the corresponding dried droplet nuclei, and (iv) the inhomogeneity of infection spreading. Furthermore, in this paper, we generalize the infection rate constant equation to account for transmission by any expiratory event. To that end, a generalized reaction mechanism that accounts for different modes of infection transmission as well as different forms of expiratory events is presented. This is followed by a comprehensive modeling of the individual infection rate constants, following which we arrive at an overall infection rate constant. In view of the above discussion, the basic reaction mechanism of Ref. 29 could be generalized to a comprehensive one where almost all possible expiratory events and modes of transmission could be included to yield

$$\begin{array}{c} S+I\overset{k_{1,\alpha \beta }}{- \to }E+I,\;\left[R1_{\alpha \beta }\right]\\ E\overset{k_{2}}{- \to }I,\;\left[R2\right]\\ I\overset{k_{3}}{- \to }0.97R+0.03D.\;\left[R3\right] \end{array}$$

In [*R*1_*αβ*_], *α* varies over different expiratory events, namely, breath, cough, sing, sneeze, and talk, while *β* varies over different modes of transmission, namely, droplet, droplet nucleus, and fomite. Thus, [*R*1_*αβ*_] essentially represents 15 reactions. The rate constants of these individual reactions are defined in Table I.

**TABLE I.** Infection rate constants for different expiratory events and modes of transmission.

*k*_1,*αβ*_	Droplet	Nucleus	Fomite
Breath	*k*_1,*bd*_	*k*_1,*bn*_	*k*_1,*bf*_
Cough	*k*_1,*cd*_	*k*_1,*cn*_	*k*_1,*cf*_
Sing	*k*_1,*gd*_	*k*_1,*gn*_	*k*_1,*gf*_
Sneeze	*k*_1,*sd*_	*k*_1,*sn*_	*k*_1,*sf*_
Talk	*k*_1,*td*_	*k*_1,*tn*_	*k*_1,*tf*_

Here, each of the parameters should be obtained for the respective combination of *α*, *β*. Including [*R*2] and [*R*3], in total, there are 15 + 2 = 17 reactions to be included in a complete model. As such, further granularity could be added by adding a location parameter *γ*. In that case, we can have *k*_1,*αβγ*_, where *γ* could represent home, school, office, transport, restaurants, and so on. In this paper, we will only consider two selected transmission modes: cough-droplets and cough-dried droplet nuclei with the rate constants *k*_1,*cd*_ and *k*_1,*cn*_, as shown in Table I. These are expected to play the more dominant roles in disease transmission. However, the approach here could be used for any other transmission routes too, with the corresponding droplet size distribution. For Covid-19, fomites are being considered as a secondary source of infection and need to be dealt separately.

Individual rate constants will allow us to delineate the different modes of transmission on average. Furthermore, the definition of individual rate constants enables quantitative investigation of the relative dominance of each mode of transmission. This constitutes one of the major goals of this paper. The infection rate constants are generalized by inclusion of the probability for infection $P_{\alpha \beta }$, averaging the collision volume over a characteristic time alongside including the dried droplet nuclei mode of transmission, in addition to the droplet mode of transmission. The revised rate constant for any expiratory event *α*, vector of transmission *β*, and location *γ* is given by the following equation:

$$k_{1,\alpha \beta \gamma }=\frac{\pi n_{total,\gamma }}{t_{c}}\int_{0}^{\tau }\sigma_{DS}^{2}\left(t\right)V_{DS}\left(t\right)P_{\alpha \beta }\left(t\right)dt.$$ (17)

Specifically, by utilizing Eqs. (3) and (5), the rate constant for the droplet mode of transmission *d* ejected during any expiratory event *α* could be calculated as

$$k_{1,\alpha d\gamma }=\frac{\pi n_{total,\gamma }}{t_{c}}\int_{0}^{\tau_{d}}\sigma_{DS}^{2}\left(t\right)V_{DS}\left(t\right)P_{\alpha d}\left(t\right)dt.$$ (18)

For the droplet nuclei, Eqs. (4) and (5) yield the rate constant *k*_1,*αnγ*_,

$$k_{1,\alpha n\gamma }=\frac{\pi n_{total,\gamma }}{t_{c}}\int_{0}^{\infty }\sigma_{DS}^{2}\left(t\right)V_{DS}\left(t\right)P_{\alpha n}\left(t\right)dt.$$ (19)

Note that we introduced a new parameter *ψ*(*t*) to calculate $P_{\alpha \beta }$, which denotes the fraction of the infectious virion population active within the dried droplet nuclei population at time *t*. As *t* → ∞, *ψ*(*t*) → 0. Thus, the integration is performed by up to about $max\left(t_{evap}\right)∼O\left(1000s\right)$—the largest evaporation time of the droplet set considered. The details on the survivability of specific SARS-CoV-2 inside dried droplet nuclei are not known. Hence, for now, we will assume that *ψ*(*t*) is independent of *d* and *n*, except when we will estimate its sensitivity in specific cases. According to the reaction mechanism given by [*R*1_*αβ*_], [*R*2], [*R*3], *E* is formed by several parallel pathways. Therefore, the corresponding rate constants become additive. Hence, the location (*γ*) dependent infection rate constant can be defined as

$$k_{1,\gamma }=\underset{\alpha ,\beta }{\sum }k_{1,\alpha \beta \gamma }.$$ (20)

While the rate constant *k*_1,*γ*_ is derived from first-principles, it still results in the same infection rate constant for a given set of ambient temperature *T*_*∞*_, *RH*_*∞*_, and population density. As shown by Lloyd-Smith *et al.*,^52^ the individual infectiousness distribution around the basic reproduction number is highly skewed. This suggests that a small fraction of infected individuals (superspreaders) are responsible for a large number of infections. Hence, the final challenge of this modeling effort is to include this effect. Such “superspreading” events could be results of (i) high local population density *n*_*total*,*γ*_, (ii) highly mobile infected individuals, and (iii) most importantly, high viral loading of the ejected respiratory droplets *ρ*_*v*_. We will see that large viral loading *ρ*_*v*_ = *ρ*_*v*,*max*_ leads to very high infection probability, which would lead to large *k*_1,*γ*_. Since the rate constant is directly proportional to *n*_*total*,*γ*_, its effect is understandable. Thus, the effect resulting from mobility needs to be accounted.

Understanding and modeling human mobility at both the individual level and the population level have garnered recent interest. See a recent review by Barbosa *et al.*^53^ for a detailed exposition on this topic. Kölbl and Helbing^54^ used the statistical data of the UK National Travel Surveys collected for 26 years by the Social Survey Division of the Office of Population Census and Surveys to arrive at a generalized distribution of human daily travel behavior. They showed that for different modes of transport *i* ranging from walking, cycling, car driving, and so on, the travel time *t*_*t*_ normalized by the average travel time for the corresponding mode of travel ${\bar{t}}_{t,i}$ and defined as $\tau_{t,i}=t_{t}/{\bar{t}}_{t,i}$, a common distribution for *τ*_*t*,*i*_, irrespective of the mode of transport could be obtained. This was also argued from an energy point of view, where $E_{i}/Ē=\tau_{t,i}$; Ē=615 kJ per person per day—the average travel energy budget of the human body according to Ref. 54. In any case, the pdf, $g_{\tau_{t}}$, after dropping the *i* given its universality is

$$g\left(\tau_{t}\right)=N^{\prime }\text{exp}\left(-\alpha /\tau_{t}-\tau_{t}/\beta \right).$$ (21)

The following constants were provided: $N=N^{\prime }/Ē=2.5,\alpha =0.2,\beta =0.7$ for the universal curve.^54^

Given two infected people *I*, it is reasonable to expect that the one with the higher mobility has more chance to infect others since they have greater exposure to the population and can infect people at different locations, all other conditions remaining fixed. Therefore, we can assume that the final infection rate constant should be proportional to *τ*_*t*_.

The corresponding infection rate constant summed over all possible types of expiratory event *α*, transmission mode *β*, and location *γ* is, thus, given by

$$k_{1,\tau_{t}}=\tau_{t}\underset{\alpha ,\beta ,\gamma }{\sum }k_{1,\alpha \beta \gamma }.$$ (22)

Clearly, *k*_1_ is now a function of two random variables *τ*_*t*_, *n*_*total*,*γ*_, and the extreme individual realization of each could correspond to the superspreading events.

Finally, the average, overall infection rate constant *k*_1_ is given by

$$k_{1}=\int_{0}^{\infty }\tau_{t}g\left(\tau_{t}\right)\underset{\alpha ,\beta ,\gamma }{\sum }k_{1,\alpha \beta \gamma }d\tau_{t}=\underset{\alpha ,\beta ,\gamma }{\sum }k_{1,\alpha \beta \gamma }.$$ (23)

This is because $\int_{0}^{\infty }\tau_{t}g\left(\tau_{t}\right)d\tau_{t}=1$ and that *∑*_*α*,*β*,*γ*_*k*_1,*αβγ*_ is independent of *τ*_*t*_. In the rest of the paper, we will mostly focus on this ensemble averaged rate constant *k*_1_. With the framework established, the individual effects of mobility and population inhomogeneity could be taken up in future works.

From the reactions [*R*1_*αβ*_], [*R*2], [*R*3], we can obtain the set of ordinary differential equations of the SEIRD model, which would govern the evolution of [*S*], [*E*], [*I*], [*R*], and [D], where the rate constants appear as respective coefficients. Here, *S* denotes susceptible, *I* denotes infected, *E* denotes exposed, *R* denotes recovered, and D denotes deceased. The square brackets denote the number of the particular population type normalized by the total population, e.g., [*I*] = *n*_*I*_/*n*_*total*_,

$$\begin{array}{c} \frac{d\left[I\right]}{dt}=k_{2}\left[E\right]-k_{3}\left[I\right],\\ \frac{d\left[E\right]}{dt}=k_{1}\left[I\right]\left[S\right]-k_{2}\left[E\right],\\ \frac{d\left[R\right]}{dt}=0.97k_{3}\left[I\right],\\ \frac{d\left[D\right]}{dt}=0.03k_{3}\left[I\right],\\ \left[S\right]+\left[E\right]+\left[I\right]+\left[R\right]+\left[D\right]=1. \end{array}$$ (24)

## III. RESULTS AND DISCUSSION

**FIG. 4.**
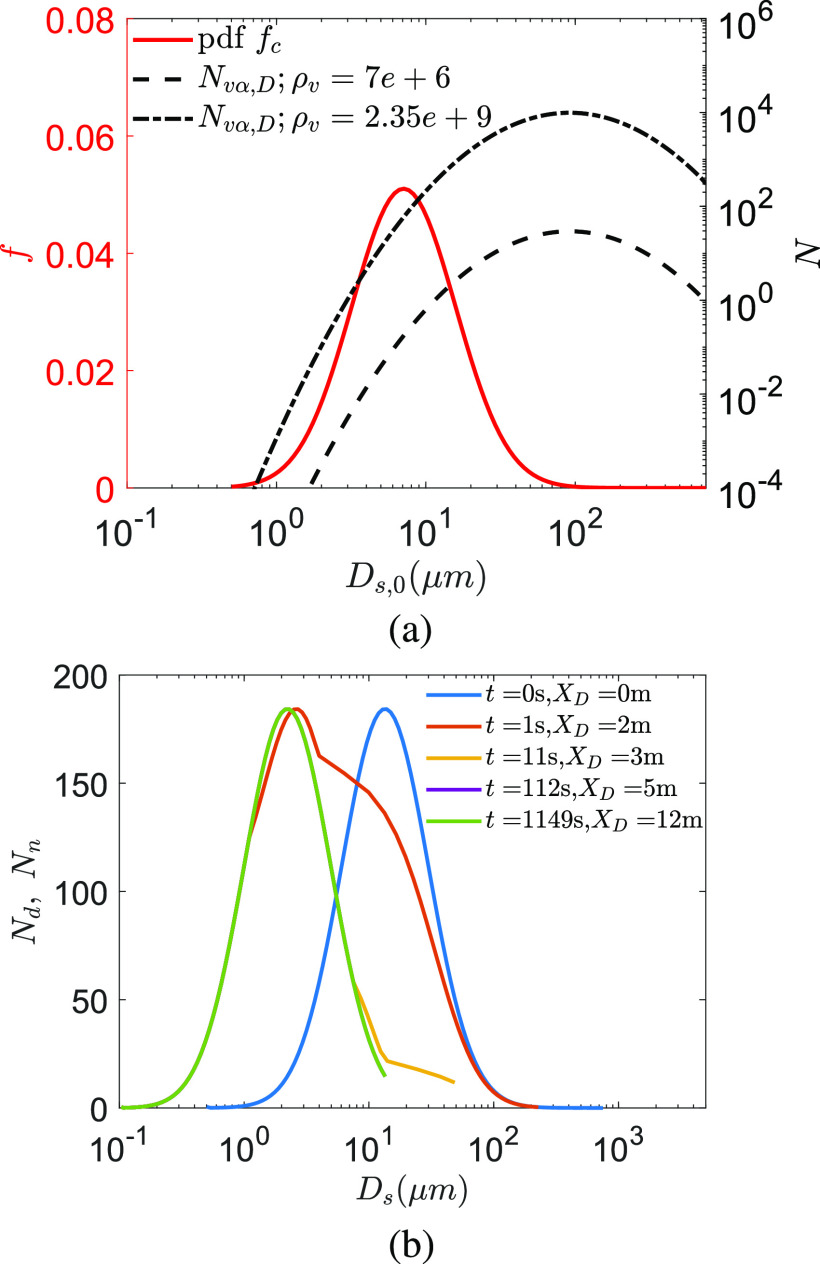
(a) Probability Density Function (PDF) *f* of droplet diameter for cough^55^ and the number of virions *N* as a function of the initial droplet size *D*_*s*,0_ at *t* = 0. Average and maximum viral load *ρ*_*v*_ = 7 × 10^6^ copies/ml and *ρ*_*v*_ = 2.35 × 10^9^ copies/ml of SARS-CoV-2, respectively, are assumed from Ref. 25. (b) Droplet and/or dried droplet nuclei size distributions at different time *t* and distance *X*_*D*_ from the origin of the respiratory jet for *T*_*∞*_ = 21.44 °C and *RH*_*∞*_ = 50%. The curves at the last three time instants end abruptly due to the loss of droplets due to settling beyond that particular diameter. For *t* > *τ*_*d*_ = 22.87 s or *X*_*D*_ > 3.52 m, all airborne droplets have been desiccated to the corresponding droplet nuclei. The size distribution remains invariant for *t* > *τ*_*d*_.

From the measurements by Duguid,^55^ the droplet size distribution from cough could be described using a log-normal distribution. The initial distribution *f*_*c*_ and the number of virions present in each droplet size for the average viral load *ρ*_*v*_ = 7 × 10^6^ copies/ml are shown in Fig. 4(a). Clearly, assuming a uniform *ρ*_*v*_, larger droplets can contain multiple virions. Thus, it is imperative to model the droplet evaporation and the jet spreading dynamics to know their eventual distributions. The total number of droplets ejected is 5000.^55^ Figure 4(b) shows the time evolution of the droplet number distribution as a function of instantaneous diameter *D*_*s*_. The shift of the distribution to the left, i.e., toward smaller *D*_*s*_, is an effect of evaporation. In addition, the right branch of the number distribution gets eroded due to settling of the larger droplets. At the conditions of interest, *T*_*∞*_ = 21.44 °C and *RH*_*∞*_ = 50%, the modal diameter of the droplet nuclei is 2.3 *µ*m at *t* = 1050 s starting from an initial modal diameter of 13.9 *µ*m at *t* = 0 s. Note that here, by the modal diameter, *D*_*s*_ corresponding to the peak of the histogram shown in Fig. 4(b) is referred. Interestingly, since small droplets evaporate fast, the left branch (small sizes) of the distribution shifts fast to further smaller sizes. A droplet with initial diameter *D*_*s*,0_ = 10 *µ*m is reduced to *D*_*s*_ = 1.96 *µ*m within *t* = 0.42 s. As such, for the entire droplet set, a modal diameter of 2.7 *µ*m, which is within 22% of the final modal diameter, is achieved within *t* = 1 s or within a distance of *X*_*D*_ = 1.8 m from the origin of the respiratory jet. *X*_*D*_ denotes the distance of the center of the respiratory jet/puff (with a diameter of *σ*_*D*_) from its origin. Within *t* = 10.6 s, *X*_*D*_ = 2.88 m, the droplet size distribution is very close to the final distribution. Due to this sharp reduction in droplet size due to evaporation (for *RH*_*∞*_ < 85%) combined with settling of large droplets, practically, for most of the time, the disease appears to be transmitted by droplets/nuclei of instantaneous diameter less than 10 *µ*m, the most probable instantaneous diameter being between 2.14 *µ*m and 2.7 *µ*m. However, it is to be noted that this diameter could be 5–6 times smaller than the initial ejected diameter of the droplet *D*_*s*,0_. In a viewpoint article, Fennelly^56^ reported that for most respiratory infections, the smaller droplets (<5 *µ*m and collected at a finite distance from the origin of the respiratory spray) were found to be pathogenic. Furthermore, Chia *et al.*^57^ reported PCR (Polymerase Chain Reaction) positive SARS-CoV-2 particles with sizes >4 *µ*m and also between 1 *µ*m and 4 *µ*m from air samples collected. Thus, our results appear to be consistent with these clinical research findings.

**FIG. 5.**
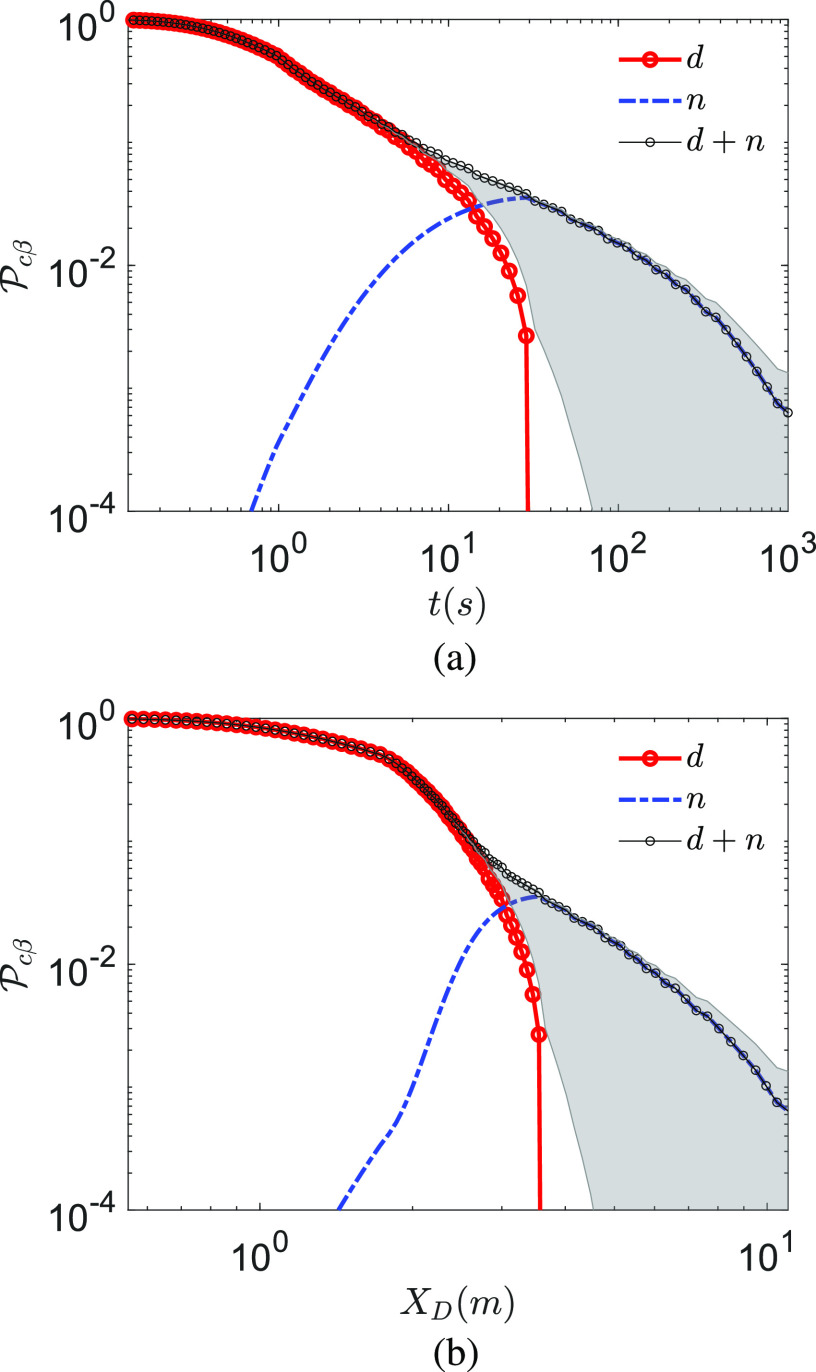
Probability of infection $P_{c\beta }$ for droplet route *d* and dried droplet nuclei route *n*, as well as total probability $P_{\beta }$ for (a) as a function of time measured from the instant of the beginning of the expiratory event and (b) as a function of distance measured from the location of the origin of the expiratory event along the center of the jet/puff trajectory. *T*_*∞*_ = 21.44 °C and *RH*_*∞*_ = 50%. The bold lines represent *ρ*_*v*_ = 7 × 10^6^ copies/ml with $t_{d\frac{1}{2}}=t_{n\frac{1}{2}}=t_{\frac{1}{2}}$, where $t_{\frac{1}{2}}=15.25$ min. The gray shaded region denotes the lower limit $t_{n\frac{1}{2}}=0.01\;t_{d\frac{1}{2}}$ and upper limit $t_{n\frac{1}{2}}=100\;t_{d\frac{1}{2}}$, respectively, with $t_{d\frac{1}{2}}=t_{\frac{1}{2}}$.

Next, we analyze the time varying infection probability. Interestingly, in Fig. 5(a), at *T*_*∞*_ = 21.44 °C and *RH*_*∞*_ = 50% for *t* > 1 s, the total probability of infection scales as $P_{c}∼t^{-2/3}$ for droplets and dried droplet nuclei. This is a combined effect of droplet evaporation, virus decay, and dilution due to the entrainment of fresh air within the jet/puff, the diameter of which increases initially as *t*^1/2^ and then as *t*^1/4^, respectively. After the droplets evaporate, the decay of the infection probability for the dried droplet nuclei slows down with respect to their droplet predecessors. This is because, while the infection probability decay for droplets is due to evaporation, settling, and dilution, the probability decay due to nuclei is due to only dilution and finite virus lifetime. Here, we are considering a very large, poorly ventilated indoor space, such as a shopping mall or a conference center, with a large number of occupants. It is to be noted that the dilution effect is arrived with the assumption that all people are in motion, but their motion do not affect the cloud aerodynamics. In reality, such motion could lead to increased turbulence and mixing, resulting in further dilution. Therefore, the increase in *σ*_*D*_ and the decay of the probability of infection could be faster in reality. An interesting feature in the original situation of interest (very large space) is that $P_{cn}$ does not continuously follow $P_{cd}$ after all the droplets evaporate. There is an accumulation of the droplet nuclei due to the evaporation of smaller droplets beforehand leading to a small jump in $P_{cn}$ at *τ*_*d*_. Indeed, the overall probability $P_{c}$ by Eq. (6) decreases without any discontinuity. From Fig. 5(b), we find that for *X*_*D*_ > 2 m, the overall probability decreases as $X_{D}^{-3}$ justifying the necessity of social distancing. However, $P_{c}<0.01$only after about 5 m.

**FIG. 6.**
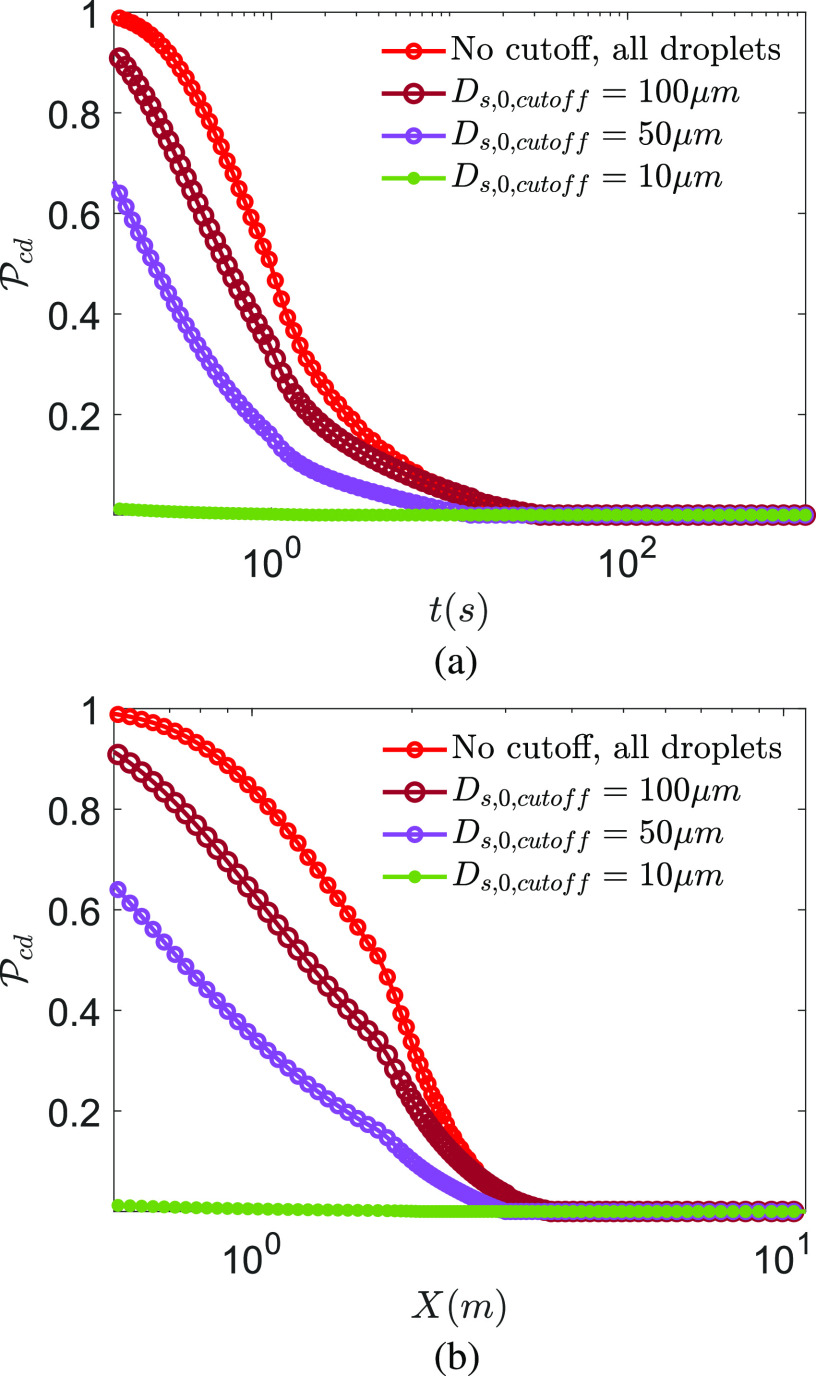
Probability of infection $P_{cd}$ for droplet route *d* at different cutoff droplet sizes (implying no droplets beyond that size) for (a) as a function of time measured from the instant of the beginning of the expiratory event and (b) as a function of distance measured from the location of the origin of the expiratory event along the center of the jet/puff trajectory. *T*_*∞*_ = 21.44 °C and *RH*_*∞*_ = 50%. *ρ*_*v*_ = 7 × 10^6^ copies/ml with $t_{d\frac{1}{2}}=t_{n\frac{1}{2}}=t_{\frac{1}{2}}$, where $t_{\frac{1}{2}}=15.25$ min.

The effect of preventing ejection of droplets beyond particular initial sizes on $P_{cd}$ is examined in Figs. 6(a) and 6(b) as a function of time and distance. For *D*_*s*,0,*cutoff*_ = 50 *µ*m, an infection probability of 0.6 is obtained for *t* → 0, suggesting droplets with *D*_*s*,0_ < 50 *µ*m are slightly more responsible for infection, at all times, for the conditions under consideration than their *D*_*s*,0_ > 50 *µ*m counterparts. This trend continues until *τ*_*d*_ when all airborne droplets evaporate. This is qualitatively consistent with the exposure analysis and results of Chen *et al.*,^58^ who considered the dispersion and evaporation of water droplets with size distribution from Duguid.^55^ However, when *D*_*s*,0,*cutoff*_ = 10 *µ*m, the corresponding probability of infection is very small, suggesting that for the average viral loading, at early times, the droplets of initial diameter 10 *µ*m < *D*_*s*,0_ < 50 *µ*m are the most lethal in terms of their probability to infect. However, while they infect, their diameters are substantially smaller.

**FIG. 7.**
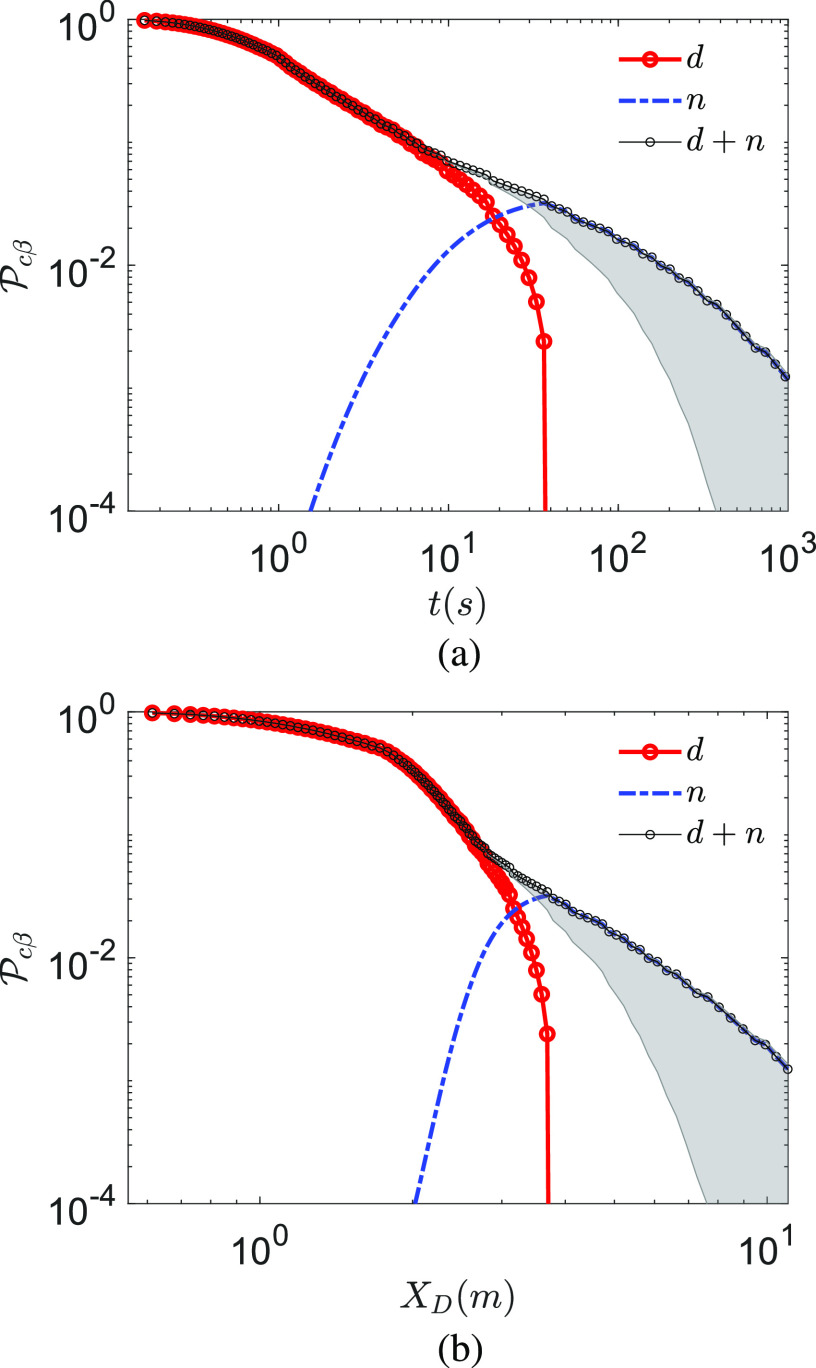
Probability of infection $P_{c\beta }$ for droplet route *d* and dried droplet nuclei route *n* (a) as a function time measured from the instant of the beginning of the expiratory event and (b) as a function of distance measured from the location of the origin of the expiratory event along the center of the jet/puff trajectory. *T*_*∞*_ = 10 °C, *RH*_*∞*_ = 20%, and *ρ*_*v*_ = 7 × 10^6^ copies/ml.

$P_{c\beta }$ at *T*_*∞*_ = 10 °C and *RH*_*∞*_ = 20% is shown in Figs. 7(a) and 7(b) as a function of time and distance. In comparison to the previous case, here, the droplet survives longer due to lower temperature, while droplet nuclei induce higher $P_{cn}$ due to longer virus half-life. Thus, at lower temperatures, the higher infection probability could be expected. However, in both cases, at short time and distance from the expiratory event, droplets (both small and large) dominate transmission, and only after most droplets evaporate, the dried droplet nuclei route is significantly activated. While the transmission probability by dried droplet nuclei is always lower than that by droplets, their lifetime is theoretically infinite as opposed to the finite lifetime *τ*_*d*_ of the droplets. Hence, despite their low instantaneous probability of infection, cumulatively, they contribute significantly, assuming that the virus remains infectious for significant times within the dried droplet nuclei. If so, as will be shown below, the persistent dried droplet nuclei appear to be a major transmission mode of the virus. It is to be recognized that these results were obtained with average viral loading *ρ*_*v*_ = 7 × 10^6^ copies/ml. If we consider *ρ*_*v*,*max*_ = 2.35 × 10^9^ copies/ml, $P_{c\beta }$ does not decay from the maximum fixed value of 1 until from about 100 s or from 5 m from the origin of the respiratory jet, along the center of the jet. Thus, it is expected that such a kind of viral loading could infect a large number of *S* potentially leading to a superspreading event.

Using $P_{\alpha \beta }\left(t\right)$ thus obtained, we can evaluate the corresponding rate constants for *d* and *n* using Eqs. (18) and (19), respectively. These rate constants for *ρ*_*v*_ = 7 × 10^6^ copies/ml are presented in Table II for four cases IA, IB, IC, and II. Cases I(A–C) correspond to *T*_*∞*_ = 21.44 °C and *RH*_*∞*_ = 50%, while case II represents *T*_*∞*_ = 10 °C and *RH*_*∞*_ = 20%. The population density in both cases is assumed to be 10 000 people/km^2^. In all cases, homogeneous mixing is assumed without any social distancing or lockdown. In all cases, *k*_1,*cnγ*_ > *k*_1,*cdγ*_. Case IA represents no restriction and clearly high rate constant values are attained in this case. Case IB represents a hypothetical situation where the ejection of all droplets with *D*_*s*,0_ > 10 *µ*m is restricted. This is hypothetically possible by stringent enforcement of population wide usage of ordinary face-masks without any exceptions. Furthermore, using *k*_1,*α*_ and *k*_3_, we can define the basic reproduction number $R_{0,\alpha }=k_{1,\alpha }/\left(0.97k_{3}\right)$. The calculated *k*_1,*cβ*_ and $R_{0,c}$ could be found in Table II. A very interesting $R_{0,c}$ trend emerges between cases IA and IB. We find that if the ejection of droplets even beyond 10 *µ*m could be completely prevented, $R_{0,c}$ drops from 4.22 (0.33, 5.63) for case IA to 0.048 for case IB. For case 1A, the numbers in the brackets denote $R_{0,c}$ for the lower limit $t_{n\frac{1}{2}}=0.01t_{d\frac{1}{2}}$ and upper limit $t_{n\frac{1}{2}}=100t_{d\frac{1}{2}}$, respectively. $R_{0,c}$ between cases 1A and 1B represent a two order of magnitude difference, and for $R_{0,c}$ at case IB, no outbreak is possible. The bifurcation point $R_{0,c}\approx 1$ is attained for the critical droplet size *D*_*s*,0_ = 27 *µ*m. This is shown in Table II as case IC. The implication is that preventing ejection of droplets with the initial size beyond 27 *µ*m would just prevent the outbreak. Of course, it is to be recognized that we are only considering cough as the mode of droplet ejection alongside many idealizing assumptions. Furthermore, these results were arrived at with the average viral load *ρ*_*v*_ = 7 × 10^6^ copies/ml. If we consider the maximum reported viral load *ρ*_*v*,*max*_ = 2.35 × 10^9^ copies/ml with free mixing among *I* and *S*, $R_{0,c}=634.12\;\left(16.32,869.28\right)$, indicating a superspreading event. As such, it could be a combination of high mobility and large viral loading of *I*, which could lead to a superspreader.

**TABLE II.** Calculated infection rate constant values for different modes of transmission for coughing with and without mask at typical indoor conditions. Cases IA, IB, and IC: *T*_*∞*_ = 21.1 °C and *RH*_*∞*_ = 50%. Case II: *T*_*∞*_ = 10 °C and *RH*_*∞*_ = 20%. For all cases, *ρ*_*v*_ = 7 × 10^6^ copies/ml.

Case	Condition	*k*_1,*cdγ*_	*k*_1,*cnγ*_	*R*_0,*c*_
IA	Cough, no mask	0.0182	0.2743	4.2219
IB	Cough, *D*_*s*,0_ = 10 *µ*m	1.58 × 10^−5^	0.0033	0.0476
	cutoff mask for all			
IC	Cough, *D*_*s*.0_ = 27 *µ*m	6.23 × 10^−4^	0.0686	0.9981
	cutoff mask for all			
II	Cough, no mask	0.0229	0.3519	5.4087

**FIG. 8.**
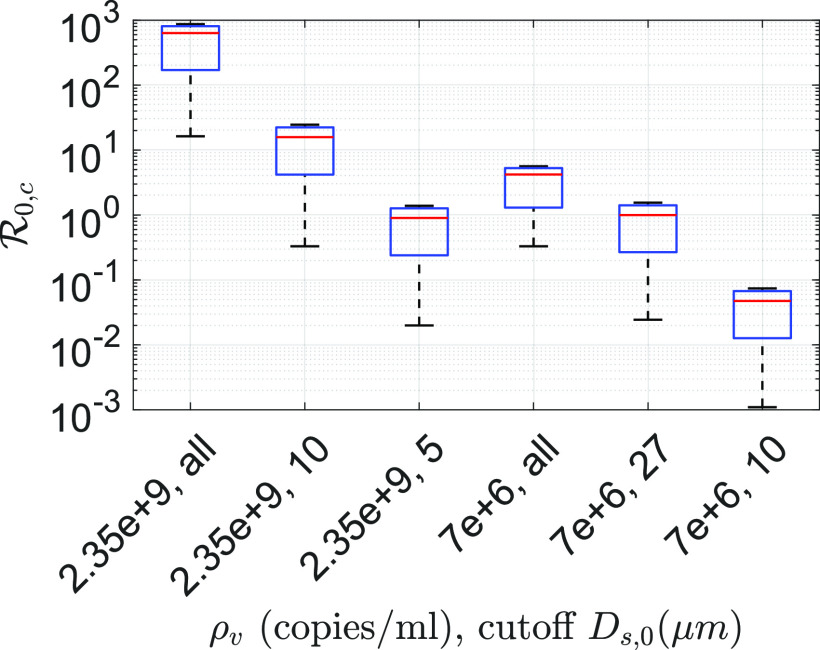
Comparison of $R_{0,c}$ for different conditions based on two different viral loadings *ρ*_*v*,*maximum*_ = 2.35 × 10^9^ copies/ml and *ρ*_*v*,*average*_ = 7 × 10^6^ copies/ml and if the ejection of droplets beyond the specified cutoff sizes is prevented. In each box, the red line denotes the equal half-life condition $t_{n\frac{1}{2}}=t_{d\frac{1}{2}}$, irrespective of the phase. The lower and upper limit corresponds to $t_{n\frac{1}{2}}=0.01\;t_{d\frac{1}{2}}$ and $t_{n\frac{1}{2}}=100\;t_{d\frac{1}{2}}$, respectively. All data are obtained at *T*_*∞*_ = 21.44 °C and *RH*_*∞*_ = 50% and with *r*_*v*_ = 0.5 implying a minimum infectious dose of 10 virions.

**FIG. 9.**
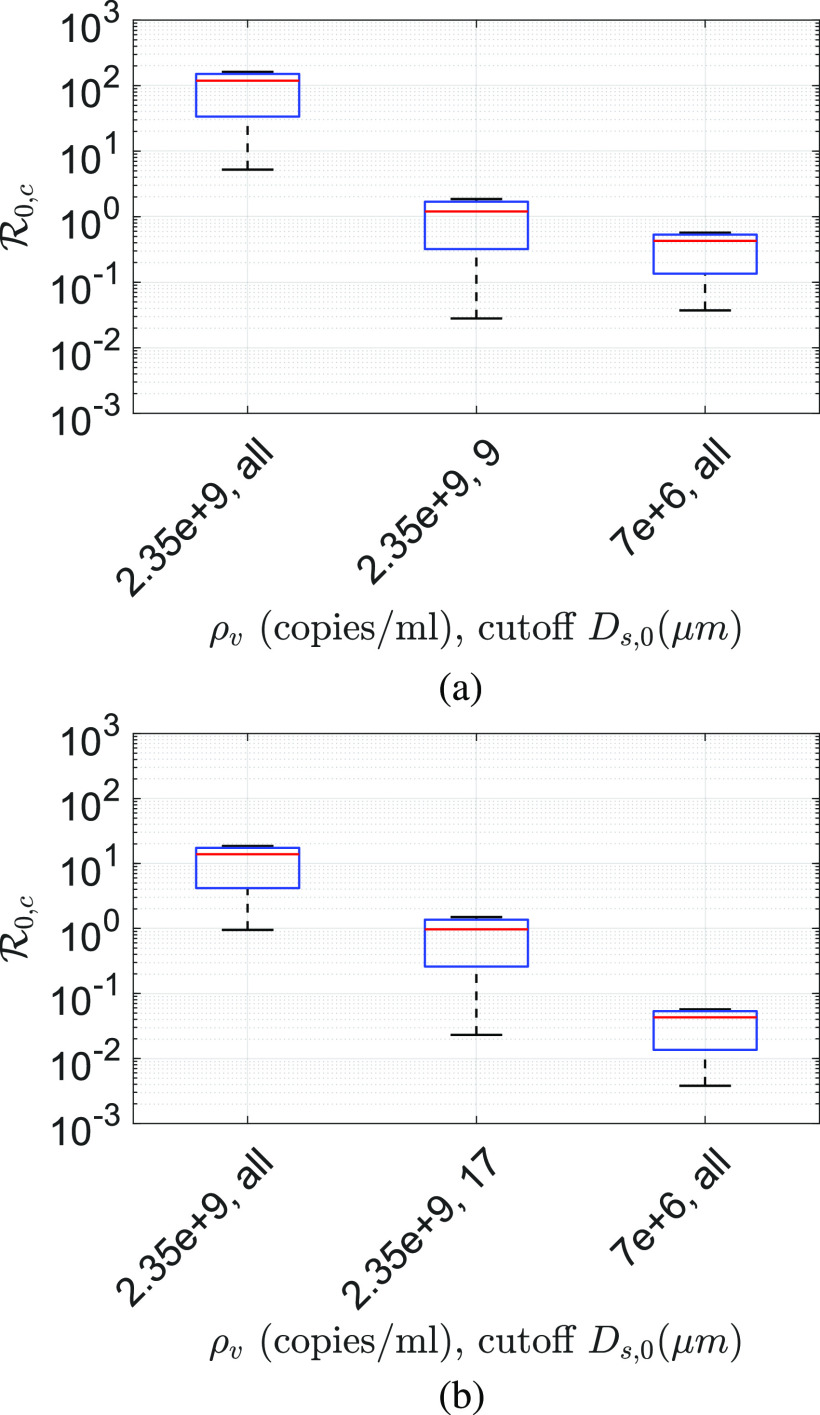
Comparison of $R_{0,c}$ for different conditions based on two different viral loadings *ρ*_*v*,*maximum*_ = 2.35 × 10^9^ copies/ml and *ρ*_*v*,*average*_ = 7 × 10^6^ copies/ml and if the ejection of droplets beyond the specified cutoff sizes is prevented. In each box, the red-line denotes the equal half-life condition $t_{n\frac{1}{2}}=t_{d\frac{1}{2}}$, irrespective of the phase. The lower and upper limit corresponds to $t_{n\frac{1}{2}}=0.01\;t_{d\frac{1}{2}}$ and $t_{n\frac{1}{2}}=100\;t_{d\frac{1}{2}}$, respectively. All data are obtained at *T*_*∞*_ = 21.44 °C and *RH*_*∞*_ = 50% and with (a) *r*_*v*_ = 0.05 implying a minimum infectious dose of 100 virions and (b) *r*_*v*_ = 0.005 implying a minimum infectious dose of 1000 virions.

$R_{0,c}$ calculated at the average and maximum viral loading *ρ*_*v*_ = 7 × 10^6^ copies/ml and *ρ*_*v*,*max*_ = 2.35 × 10^9^ copies/ml, respectively, for different droplet size cutoffs at *T*_*∞*_ = 21.44 °C and *RH*_*∞*_ = 50% is shown in Fig. 8. The cutoff *D*_*s*,0_ means that all droplets with sizes *D*_*s*,0_ > *D*_*s*,0,*cutoff*_ are prevented from ejecting. Figure 8 also shows the sensitivity of the assumption $t_{n\frac{1}{2}}=t_{d\frac{1}{2}}=t_{\frac{1}{2}}$ on the results. In Fig. 8, the lower and upper limits represent the conditions $t_{n\frac{1}{2}}=0.01\;t_{d\frac{1}{2}}$ and $t_{n\frac{1}{2}}=100\;t_{d\frac{1}{2}}$, respectively. If all droplets are allowed to be ejected at average viral loading, for $t_{n\frac{1}{2}}=0.01\;t_{d\frac{1}{2}}$, $R_{0,c}=0.33$, while for $t_{n\frac{1}{2}}=100\;t_{d\frac{1}{2}}$, $R_{0,c}=5.63$ with the base $R_{0,c}=4.22$ for the typical indoor conditions assumed above. Clearly, the change in the lower limit of $t_{n\frac{1}{2}}$ is much more sensitive than its upper limit. This is because even if the viral lifetime is much longer, dilution reduces infection probability. However, with *ρ*_*v*,*max*_ = 2.35 × 10^9^ copies/ml for $t_{n\frac{1}{2}}=0.01\;t_{d\frac{1}{2}}$, $R_{0,c}=16.32$, while for $t_{n\frac{1}{2}}=100\;t_{d\frac{1}{2}}$, $R_{0,c}=869.28$ around the base case of $R_{0,c}=634.12$, all other conditions remaining the same. Interestingly, with *D*_*s*,0,*cutoff*_ = 10 *µ*m, $R_{0,c}$ reduces by a factor of 40 with respect to no cutoff condition. However, *D*_*s*,0,*cutoff*_ = 5 *µ*m reduces $R_{0,c}$ by another factor of 18 with respect to *D*_*s*,0,*cutoff*_ = 10 *µ*m condition for the base cases. At this condition, $R_{0,c}<1$. For both viral loadings, the maximum and the averaged blocking droplets *D*_*s*,0_ ≥ 5 *µ*m can theoretically yield $R_{0,c}\leq 1$. All the results, so far, have been obtained with *r*_*v*_ = 0.5, which implies a minimum infectious dose of 10 virions. Figures 9(a) and 9(b) show the corresponding $R_{0,c}$ for *r*_*v*_ = 0.05 and *r*_*v*_ = 0.005, respectively. These imply minimum infectious doses of 100 and 1000 virions, respectively. While the qualitative trend is similar, indeed, $R_{0,c}$ for these two cases are much lower in comparison to *r*_*v*_ = 0.5. As such, it seems likely that the minimum infectious dose of SARS-Cov-2 is O(10). While the base $R_{0,c}=4.22$ for the average viral loading, *r*_*v*_ = 0.5 and no cutoff is consistent with that of the reported values for Covid-19,^59^ the order of magnitude larger values of $R_{0,c}$ obtained at maximum viral loading should be viewed in the context of superspreading events.

**FIG. 10.**
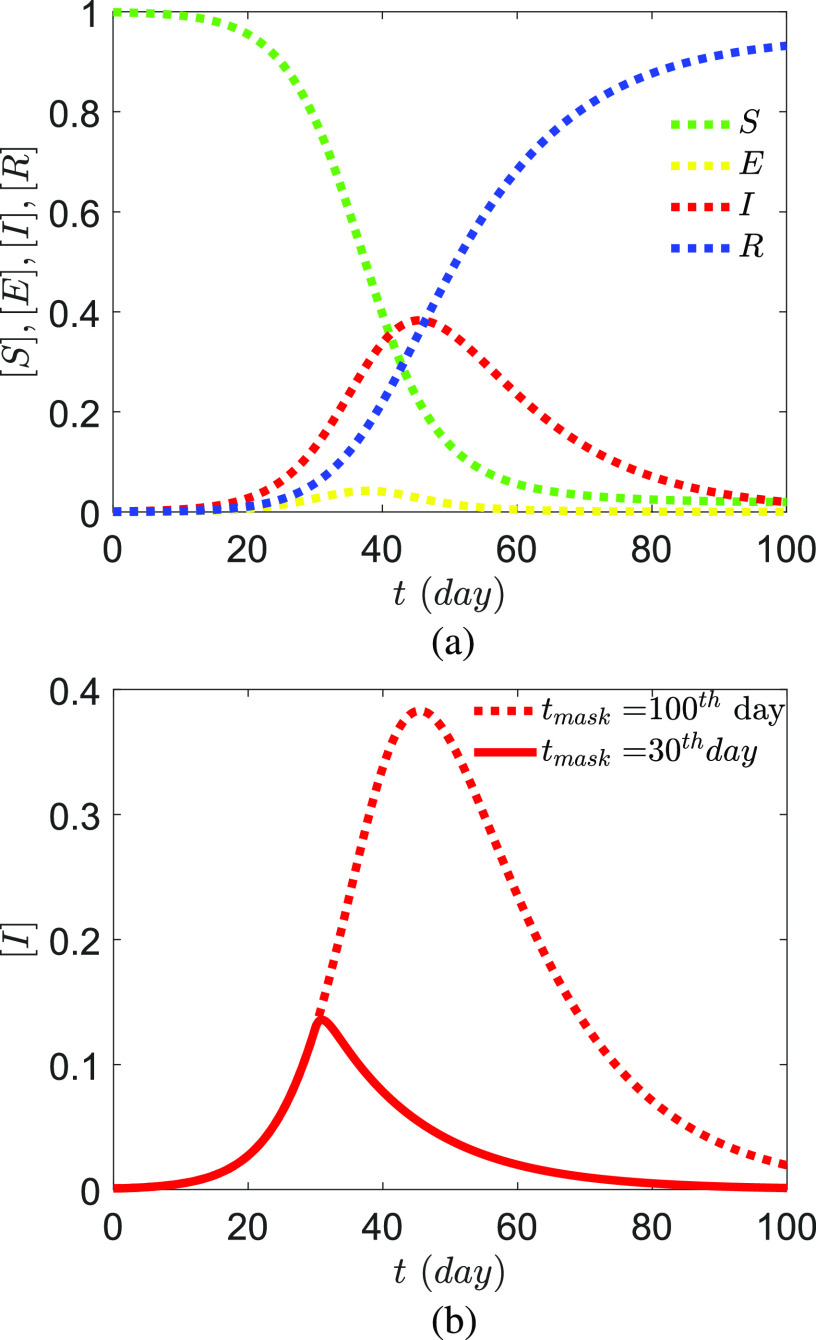
SEIRD-droplet/nuclei model output with computed rate constants mentioned in Table II for case IA. No lockdown and free mixing are assumed. (a) Case IA, no mask usage and (b) case IB, all *I* wear masks from the (i) 100th day and (ii) 30th day of the onset of the pandemic that prevents the ejection of *D*_*s*,0_ > 10 *µ*m droplets with *ρ*_*v*_ = 7 × 10^6^ copies/ml. Calculated rate constants are shown in Table II for cases IA and IB. The masks for this case are assumed to prevent the ejection of all particles greater than 10 *µ*m.

Using the governing equation (24), the evolution of the pandemic for average viral loading and *r*_*v*_ = 0.5 for case IA is presented in Fig. 10(a). The growth rate of the infected population for case IA and case IB is shown in Fig. 10(b) with the assumption that the usage of face masks for the entire *I* population (which would practically be required for the entire population) is implemented after a fixed time from the onset of the outbreak. The effectiveness of facemasks has been mechanistically proven.^60,61^ As expected, in this SEIRD model with *ab initio* infection rate constants, the effect of the usage of masks is almost immediate since the assumed latency period is only one day. Leffler *et al.*^62^ analyzed Covid-19 data from 198 countries to conclude that government policies on mask wearing significantly reduced mortality. A significant feature of the results of this paper is that, while they are computed mechanistically from first-principles with assumptions and limitations, they produce physically meaningful outcomes.

## IV. CONCLUSIONS

Current epidemiological models for infectious respiratory diseases do not account for the underlying flow physics of transmission. This work presents a SEIRD model developed from the mechanics of respiratory disease transmission, including but not limited to known SARS-CoV-2 virus characteristics, thermodynamics, transport, and aerodynamics of respiratory droplets/nuclei cloud leading to its interaction with a susceptible population. Starting with a well known cough droplet size distribution, we derived the time dependent probability of infection and infection rate constants for different routes accounting for viral load, virus stability, respiratory droplet cloud aerodynamics, evaporation, and crystallization for poorly ventilated conditions. Assumptions are inherent in all models. While the assumptions pertaining to the present model have been described throughout the paper at their respective points of discussion, for the sake of clarity, we revisit the three major model assumptions here. (1) Infectious respiratory droplets contain water, electrolytes, mucus, enzymes, and virus. Here, we model it using 1% NaCl–water droplets, thereby accounting for the first and second major components of the mucosalivary fluid. The evaporation characteristics are well modeled, as shown in Fig. 4. Viral load is assumed to be uniform across all droplet sizes. (2) The droplets are ejected in a turbulent cough jet, which transitions to a puff in a poorly ventilated, quiescent, large indoor space. Typical droplet size distribution, mean thermodynamic, and aerodynamic state of such jets and puffs are considered in which the droplets evaporate until their desiccation. While the results only reflect the cough jet and its infection dynamics, the model could be easily extended to account for similar respiratory jets. We do not consider the effect of turbulence or buoyancy on droplet evaporation or in mixing of the aerosols. The internal air-circulation effect has not been considered since it would be location specific. (3) Infection occurs when a certain number of virions are inhaled upon collision of susceptible individuals with the respiratory puff. The collision frequency combined with probability of infection yields the rate constants for the diseases spread ordinary differential equations. The indoor space volume considered is much larger than the puff volume over the time considered. However, the model could be easily extended to a situation where the indoor space is filled with the aerosols.

Considering a well known cough droplet size distribution, most number of droplets have an initial diameter of *D*_*s*,0_ = 13.9 *µ*m, but within 1 s of their ejection, most number of droplets of the same set gets reduced to a diameter of 2.7 *µ*m due to evaporation, accounting for the thermodynamic state of the exhaled air, at typical, air-conditioned yet quiescent indoor space. For the average viral loading, at early times, the droplets of initial diameter 10 *µ*m < *D*_*s*,0_ < 50 *µ*m are the most lethal in terms of their probability to infect. However, while they infect, their diameters could be 5–6 times smaller. Indeed, for most of the time, infection is spread by inhalation of small, airborne droplets or their desiccated nuclei. While the instantaneous probability of infection by droplets is significantly larger than its dried nuclei in the short time and range, the much longer persistence of the dried nuclei results in its stronger relative contribution to the infection rate constant, under the assumption that the virus half-life is independent of the phase of its vector. The infection rate constant is derived *ab initio* by calculating collision frequency between the droplets/nuclei cloud and the susceptible population for different ambient conditions including the probability of infection. The SEIRD model output obtained with the calculated rate constants for average viral loading, for the specific conditions of interest, shows that preventing ejection of droplets with the initial diameter greater than 10 *µ*m can potentially prevent further outbreaks even for a minimum infectious dose of 10 virions. The critical droplet diameter, preventing ejection of droplets above which would result in $R_{0,c}\approx 1$, is found to be 27 *µ*m. For maximum viral loading, the critical droplet diameter is 5 *µ*m to just prevent the outbreaks. Furthermore, the strong sensitivity of $R_{0,c}$ on the variation of virus half-life at different phases of the droplet/aerosol as well as the minimum infectious dose is demonstrated.

## DATA AVAILABILITY

The data that support the findings of this study are available from the corresponding author upon reasonable request.

## References

[1] L. Morawska and D. K. Milton, “It is time to address airborne transmission of coronavirus disease 2019 (COVID-19),” Clin. Infect. Dis. (published online 2020). 10.1093/cid/ciaa939 PMC7454469 32628269

[2] Y. Liu, Z. Ning, Y. Chen, M. Guo, Y. Liu, N. K. Gali, L. Sun, Y. Duan, J. Cai, D. Westerdahl *et al.*, “Aerodynamic analysis of SARS-CoV-2 in two Wuhan hospitals,” Nature 582, 557 (2020). 10.1038/s41586-020-2271-3 32340022

[3] World Health Organization, “Transmission of SARS-CoV-2: Implications for infection prevention precautions, scientific brief 9 july 2020,” Technical Report No. WHO/2019-nCoV/Sci_Brief/Transmission_modes/2020.3, World Health Organization, 2020.

[4] R. Mittal, R. Ni, and J.-H. Seo, “The flow physics of COVID-19,” J. Fluid Mech. 894, F2 (2020). 10.1017/jfm.2020.330

[5] G. Busco, S. R. Yang, J. Seo, and Y. A. Hassan, “Sneezing and asymptomatic virus transmission,” Phys. Fluids 32, 073309 (2020). 10.1063/5.0019090 PMC7367211 32684746

[6] T. Dbouk and D. Drikakis, “On coughing and airborne droplet transmission to humans,” Phys. Fluids 32, 053310 (2020). 10.1063/5.0011960 PMC7239332 32574229

[7] S. Balachandar, S. Zaleski, A. Soldati, G. Ahmadi, and L. Bourouiba, “Host-to-host airborne transmission as a multiphase flow problem for science-based social distance guidelines,” Int. J. Multiphase Flow 132, 103439 (2020). 10.1016/j.ijmultiphaseflow.2020.103439

[8] R. Mittal, C. Meneveau, and W. Wu, “A mathematical framework for estimating risk of airborne transmission of COVID-19 with application to face mask use and social distancing,” Phys. Fluids 32, 101903 (2020). 10.1063/5.0025476 PMC7583361 33100806

[9] H. Li, F. Y. Leong, G. Xu, Z. Ge, C. W. Kang, and K. H. Lim, “Dispersion of evaporating cough droplets in tropical outdoor environment,” Phys. Fluids 32, 113301 (2020). 10.1063/5.0026360 PMC7685245 33244215

[10] M. A. Kanso, J. H. Piette, J. A. Hanna, and A. J. Giacomin, “Coronavirus rotational diffusivity,” Phys. Fluids 32, 113101 (2020). 10.1063/5.0031875 PMC7641315 33162728

[11] V. Stadnytskyi, C. E. Bax, A. Bax, and P. Anfinrud, “The airborne lifetime of small speech droplets and their potential importance in SARS-CoV-2 transmission,” Proc. Natl. Acad. Sci. U. S. A. 117, 11875–11877 (2020). 10.1073/pnas.2006874117 32404416 PMC7275719

[12] J. Duguid, “The numbers and the sites of origin of the droplets expelled during expiratory activities,” Edinburgh Med. J. 52, 385–401 (1945). PMC5286249 21009905

[13] X. Xie, Y. Li, H. Sun, and L. Liu, “Exhaled droplets due to talking and coughing,” J. R. Soc., Interface 6, S703–S714 (2009). 10.1098/rsif.2009.0388.focus 19812073 PMC2843952

[14] C. Y. H. Chao, M. P. Wan, L. Morawska, G. R. Johnson, Z. D. Ristovski, M. Hargreaves, K. Mengersen, S. Corbett, Y. Li, X. Xie *et al.*, “Characterization of expiration air jets and droplet size distributions immediately at the mouth opening,” J. Aerosol Sci. 40, 122–133 (2009). 10.1016/j.jaerosci.2008.10.003 32287373 PMC7126899

[15] L. Bourouiba, E. Dehandschoewercker, and J. W. M. Bush, “Violent expiratory events: On coughing and sneezing,” J. Fluid Mech. 745, 537–563 (2014). 10.1017/jfm.2014.88

[16] L. Bourouiba, “Turbulent gas clouds and respiratory pathogen emissions: Potential implications for reducing transmission of COVID-19,” JAMA 323, 1837–1838 (2020). 10.1001/jama.2020.4756 32215590

[17] V. I. Khvorostyanov and J. A. Curry, Thermodynamics, Kinetics, and Microphysics of Clouds (Cambridge University Press, 2014).

[18] E. P. Vejerano and L. C. Marr, “Physico-chemical characteristics of evaporating respiratory fluid droplets,” J. R. Soc., Interface 15, 20170939 (2018). 10.1098/rsif.2017.0939 29491178 PMC5832737

[19] L. C. Marr, J. W. Tang, J. Van Mullekom, and S. S. Lakdawala, “Mechanistic insights into the effect of humidity on airborne influenza virus survival, transmission and incidence,” J. R. Soc., Interface 16, 20180298 (2019). 10.1098/rsif.2018.0298 30958176 PMC6364647

[20] K. Lin and L. C. Marr, “Humidity-dependent decay of viruses, but not bacteria, in aerosols and droplets follows disinfection kinetics,” Environ. Sci. Technol. 54, 1024–1032 (2019). 10.1021/acs.est.9b04959 31886650

[21] M. J. Keeling and P. Rohani, Modeling Infectious Diseases in Humans and Animals (Princeton University Press, 2011).

[22] A. L. Bertozzi, E. Franco, G. Mohler, M. B. Short, and D. Sledge, “The challenges of modeling and forecasting the spread of COVID-19,” Proc. Natl. Acad. Sci. 117, 16732–16738 (2020). 10.1073/pnas.2006520117 32616574 PMC7382213

[23] D. Adam, “Special report: The simulations driving the world’s response to COVID-19,” Nature 580, 316 (2020). 10.1038/d41586-020-01003-6 32242115

[24] C. J. E. Metcalf, D. H. Morris, and S. W. Park, “Mathematical models to guide pandemic response,” Science 369, 368–369 (2020). 10.1126/science.abd1668 32703861

[25] R. Wölfel, V. M. Corman, W. Guggemos, M. Seilmaier, S. Zange, M. A. Müller, D. Niemeyer, T. C. Jones, P. Vollmar, C. Rothe *et al.*, “Virological assessment of hospitalized patients with COVID-2019,” Nature 581, 465–469 (2020). 10.1038/s41586-020-2196-x 32235945

[26] K. E. Barrett, S. M. Barman, H. L. Brooks, and J. X.-J. Yuan, Ganong’s Review of Medical Physiology (McGraw-Hill Education, 2019).

[27] M. Schuit, S. Ratnesar-Shumate, J. Yolitz, G. Williams, W. Weaver, B. Green, D. Miller, M. Krause, K. Beck, S. Wood *et al.*, “Airborne SARS-CoV-2 is rapidly inactivated by simulated sunlight,” J. Infect. Dis. 222, 564 (2020). 10.1093/infdis/jiaa334 32525979 PMC7313838

[28] U.S. Department of Homeland Security, “Estimated airborne decay of SARS-CoV-2,” Technical Report (2020), https://www.dhs.gov/science-and-technology/sars-airborne-calculator.

[29] S. Chaudhuri, S. Basu, P. Kabi, V. R. Unni, and A. Saha, “Modeling the role of respiratory droplets in COVID-19 type pandemics,” Phys. Fluids 32, 063309 (2020). 10.1063/5.0015984 PMC7327718 32624650

[30] J. G. Allen and L. C. Marr, “Recognizing and controlling airborne transmission of SARS-CoV-2 in indoor environments,” Indoor Air 30, 557 (2020). 10.1111/ina.12697 32557915 PMC7323102

[31] C. N. Haas, “Estimation of risk due to low doses of microorganisms: A comparison of alternative methodologies,” Am. J. Epidemiol. 118, 573–582 (1983). 10.1093/oxfordjournals.aje.a113662 6637984

[32] M. Nicas, “An analytical framework for relating dose, risk, and incidence: An application to occupational tuberculosis infection,” Risk Anal. 16, 527–538 (1996). 10.1111/j.1539-6924.1996.tb01098.x 8819343

[33] G. N. Sze To, M. P. Wan, C. Y. H. Chao, F. Wei, S. C. T. Yu, and J. K. C. Kwan, “A methodology for estimating airborne virus exposures in indoor environments using the spatial distribution of expiratory aerosols and virus viability characteristics,” Indoor Air 18, 425–438 (2008). 10.1111/j.1600-0668.2008.00544.x 18691266

[34] E. C. Riley, G. Murphy, and R. L. Riley, “Airborne spread of measles in a suburban elementary school,” Am. J. Epidemiol. 107, 421–432 (1978). 10.1093/oxfordjournals.aje.a112560 665658

[35] G. Buonanno, L. Stabile, and L. Morawska, “Estimation of airborne viral emission: Quanta emission rate of SARS-CoV-2 for infection risk assessment,” Environ. Int. 141, 105794 (2020). 10.1016/j.envint.2020.105794 32416374 PMC7211635

[36] M. P. Zwart, L. Hemerik, J. S. Cory, J. A. G. M. de Visser, F. J. J. A. Bianchi, M. M. Van Oers, J. M. Vlak, R. F. Hoekstra, and W. Van der Werf, “An experimental test of the independent action hypothesis in virus-insect pathosystems,” Proc. R. Soc. B 276, 2233–2242 (2009). 10.1098/rspb.2009.0064 PMC2677602 19324752

[37] N. Abani and R. D. Reitz, “Unsteady turbulent round jets and vortex motion,” Phys. Fluids 19, 125102 (2007). 10.1063/1.2821910

[38] B. Cushman-Roisin, Environmental Fluid Mechanics (John Wiley & Sons, 2019).

[39] R. S. Scorer and R. S. Scorer, Dynamics of Meteorology and Climate (Wiley Chichester, 1997).

[40] Z. Y. Han, W. G. Weng, and Q. Y. Huang, “Characterizations of particle size distribution of the droplets exhaled by sneeze,” J. R. Soc., Interface 10, 20130560 (2013). 10.1098/rsif.2013.0560 24026469 PMC3785820

[41] W. A. Sirignano, Fluid Dynamics and Transport of Droplet and Sprays (Cambridge University Press, 2010).

[42] E. Mansour, R. Vishinkin, S. Rihet, W. Saliba, F. Fish, P. Sarfati, and H. Haick, “Measurement of temperature and relative humidity in exhaled breath,” Sens. Actuators, B 304, 127371 (2020). 10.1016/j.snb.2019.127371

[43] G. Abramovich, The Theory of Turbulent Jets (MIT Press, 2003).

[44] A. Naillon, P. Duru, M. Marcoux, and M. Prat, “Evaporation with sodium chloride crystallization in a capillary tube,” J. Cryst. Growth 422, 52–61 (2015). 10.1016/j.jcrysgro.2015.04.010

[45] H. Derluyn, “Salt transport and crystallization in porous limestone: Neutron-X-ray imaging and poromechanical modeling,” Ph.D. thesis, ETH Zurich, 2012.

[46] P. Kabi, A. Saha, S. Chaudhuri, and S. Basu, “Insights on drying and precipitation dynamics of respiratory droplets in the perspective of COVID-19,” arXiv:2008.00934 (2020). 10.1063/5.0037360 PMC7976039 33746480

[47] N. I. Stilianakis and Y. Drossinos, “Dynamics of infectious disease transmission by inhalable respiratory droplets,” J. R. Soc., Interface 7, 1355–1366 (2010). 10.1098/rsif.2010.0026 20164087 PMC2894888

[48] C. K. Law, Combustion Physics (Cambridge University Press, 2006).

[49] I. Karamouzas, B. Skinner, and S. J. Guy, “Universal power law governing pedestrian interactions,” Phys. Rev. Lett. 113, 238701 (2014). 10.1103/physrevlett.113.238701 25526171

[50] U. Weidmann, “Transporttechnik der fussgänger-transporttechnische eigenschaftendes fussgängerverkehrs (literaturstudie),” (1993), available at https://www.research-collection.ethz.ch/handle/20.500.11850/242008.

[51] J. Y. Hsu, R. A. Stone, R. B. Logan-Sinclair, M. Worsdell, C. M. Busst, and K. F. Chung, “Coughing frequency in patients with persistent cough: Assessment using a 24 hour ambulatory recorder,” Eur. Respir. J. 7, 1246–1253 (1994). 10.1183/09031936.94.07071246 7925902

[52] J. O. Lloyd-Smith, S. J. Schreiber, P. E. Kopp, and W. M. Getz, “Superspreading and the effect of individual variation on disease emergence,” Nature 438, 355–359 (2005). 10.1038/nature04153 16292310 PMC7094981

[53] H. Barbosa, M. Barthelemy, G. Ghoshal, C. R. James, M. Lenormand, T. Louail, R. Menezes, J. J. Ramasco, F. Simini, and M. Tomasini, “Human mobility: Models and applications,” Phys. Rep. 734, 1–74 (2018). 10.1016/j.physrep.2018.01.001

[54] R. Kölbl and D. Helbing, “Energy laws in human travel behaviour,” New J. Phys. 5, 48 (2003). 10.1088/1367-2630/5/1/348

[55] J. P. Duguid, “The size and the duration of air-carriage of respiratory droplets and droplet-nuclei,” Epidemiol. Infect. 44, 471–479 (1946). 10.1017/s0022172400019288 PMC2234804 20475760

[56] K. P. Fennelly, “Particle sizes of infectious aerosols: Implications for infection control,” Lancet Respir. Med. 8, 914 (2020). 10.1016/s2213-2600(20)30323-4 32717211 PMC7380927

[57] P. Y. Chia, K. K. Coleman, Y. K. Tan, S. W. X. Ong, M. Gum, S. K. Lau, X. F. Lim, A. S. Lim, S. Sutjipto, P. H. Lee *et al.*, “Detection of air and surface contamination by SARS-CoV-2 in hospital rooms of infected patients,” Nat. Commun. 11, 2800 (2020). 10.1038/s41467-020-16670-2 32472043 PMC7260225

[58] W. Chen, N. Zhang, J. Wei, H.-L. Yen, and Y. Li, “Short-range airborne route dominates exposure of respiratory infection during close contact,” Build. Environ. 176, 106859 (2020). 10.1016/j.buildenv.2020.106859

[59] Y. Liu, A. A. Gayle, A. Wilder-Smith, and J. Rocklöv, “The reproductive number of COVID-19 is higher compared to SARS coronavirus,” J. Travel Med. 27, taaa021 (2020). 10.1093/jtm/taaa021 32052846 PMC7074654

[60] S. Verma, M. Dhanak, and J. Frankenfield, “Visualizing the effectiveness of face masks in obstructing respiratory jets,” Phys. Fluids 32, 061708 (2020). 10.1063/5.0016018 PMC7327717 32624649

[61] N. H. L. Leung, D. K. W. Chu, E. Y. C. Shiu, K.-H. Chan, J. J. McDevitt, B. J. P. Hau, H.-L. Yen, Y. Li, D. K. M. Ip, J. S. M. Peiris *et al.*, “Respiratory virus shedding in exhaled breath and efficacy of face masks,” Nat. Med. 26, 676–680 (2020). 10.1038/s41591-020-0843-2 32371934 PMC8238571

[62] C. T. Leffler, E. B. Ing, J. D. Lykins, M. C. Hogan, C. A. McKeown, and A. Grzybowski, “Association of country-wide coronavirus mortality with demographics, testing, lockdowns, and public wearing of masks update July 2, 2020,” Am. J. Trop. Med. Hyg. (published online 2020). 10.4269/ajtmh.20-1015 PMC7695060 33124541

